# Efficient enzyme‐free isolation of brain‐derived extracellular vesicles

**DOI:** 10.1002/jev2.70011

**Published:** 2024-11-07

**Authors:** Andreu Matamoros‐Angles, Emina Karadjuzovic, Behnam Mohammadi, Feizhi Song, Santra Brenna, Susanne Caroline Meister, Bente Siebels, Hannah Voß, Carolin Seuring, Isidre Ferrer, Hartmut Schlüter, Matthias Kneussel, Hermann Clemens Altmeppen, Michaela Schweizer, Berta Puig, Mohsin Shafiq, Markus Glatzel

**Affiliations:** ^1^ Institute of Neuropathology University Medical Center Hamburg‐Eppendorf (UKE) Hamburg Germany; ^2^ Department of Neurology, Experimental Research in Stroke and Inflammation (ERSI) University Medical Center Hamburg‐Eppendorf (UKE) Hamburg Germany; ^3^ Section Mass Spectrometry and Proteomics University Medical Center Hamburg‐Eppendorf (UKE) Hamburg Germany; ^4^ Multi‐User‐CryoEM‐Facility Centre for Structural Systems Biology (CSSB) Hamburg Germany; ^5^ Department of Chemistry Universität Hamburg Hamburg Germany; ^6^ Leibniz Institute of Virology (LIV) Hamburg Germany; ^7^ IDIBELL University of Barcelona L'Hospitalet de Llobregat Spain; ^8^ Institute for Molecular Neurogenetics, Center for Molecular Neurobiology Hamburg (ZMNH) University Medical Center Hamburg‐Eppendorf (UKE) Hamburg Germany; ^9^ Electron Microscopy Core Facility, Center for Molecular Neurobiology (ZMNH) University Medical Center Hamburg‐Eppendorf (UKE) Hamburg Germany

**Keywords:** BDEVs, brain‐derived EVs, collagenase‐free, EV corona, GM130, prion protein, proteomics, PrP^C^

## Abstract

Extracellular vesicles (EVs) have gained significant attention as pathology mediators and potential diagnostic tools for neurodegenerative diseases. However, isolation of brain‐derived EVs (BDEVs) from tissue remains challenging, often involving enzymatic digestion steps that may compromise the integrity of EV proteins and overall functionality. Here, we describe that collagenase digestion, commonly used for BDEV isolation, produces undesired protein cleavage of EV‐associated proteins in brain tissue homogenates and cell‐derived EVs. In order to avoid this effect, we studied the possibility of isolating BDEVs with a reduced amount of collagenase or without any protease. Characterization of the isolated BDEVs from mouse and human samples (both female and male) revealed their characteristic morphology and size distribution with both approaches. However, we show that even minor enzymatic digestion induces ‘artificial’ proteolytic processing in key BDEV markers, such as Flotillin‐1, CD81, and the cellular prion protein (PrP^C^), whereas avoiding enzymatic treatment completely preserves their integrity. We found no major differences in mRNA and protein content between non‐enzymatically and enzymatically isolated BDEVs, suggesting that the same BDEV populations are purified with both approaches. Intriguingly, the lack of Golgi marker GM130 signal, often referred to as contamination indicator (or negative marker) in EV preparations, seems to result from enzymatic digestion rather than from its actual absence in BDEV samples. Overall, we show that non‐enzymatic isolation of EVs from brain tissue is possible and avoids artificial pruning of proteins while achieving an overall high BDEV yield and purity. This protocol will help to understand the functions of BDEV and their associated proteins in a near‐physiological setting, thus opening new research approaches.

## 1 INTRODUCTION

Extracellular vesicles (EVs) are a heterogenous family of nanosized lipid bilayer‐enclosed vesicular structures, which virtually all cell types secrete by evolutionarily conserved processes (De Sousa *et al*., 2023; Van Niel *et al.*, 2018). The term ‘EVs’ includes all currently known subpopulations such as exosomes, ectosomes, apoptotic EVs, and the more recently described ones such as mitovesicles or migrasomes (D'Acunzo *et al.*, 2022; Ma *et al*., 2015; Poon *et al*., 2019). These subpopulations differ in their composition/cargo and biogenesis but are largely indistinguishable by most used isolation methods, unless a further specific affinity‐based method is used to isolate them from the complex pool present in a given biological sample (Théry *et al*., 2018; Welsh *et al*., 2024). EVs contain tissue‐ and cell‐specific cargo composed of proteins (membrane‐associated or intraluminal/cytosolic), lipids, and nucleic acids such as DNA (mitochondrial DNA, single‐ and double‐stranded DNA) and RNA (mRNA and diverse non‐coding RNAs). By transferring their incorporated and exposed molecules, they elicit biological effects in (targeted) recipient cells (O'Brien *et al*., 2022; Valadi *et al*., 2007; Van Niel *et al*., 2018). Moreover, EVs are naturally surrounded by a corona of attached/associated proteins and other biomolecules that may regulate interaction with target cells (Tóth *et al.*, 2021; Wolf *et al*., 2022).

In the central nervous system (CNS), EVs have been highlighted as relevant actors in the complex intercellular communication regulating diverse aspects such as synaptic activity, circuit connectivity, and neuronal differentiation (Antoniou *et al.*, 2023; Solana‐Balaguer *et al*., 2023; Song *et al.*, 2023). However, the transfer of signals, proteins, and genetic information between brain cells via EVs is not only relevant in physiology but also in various pathological conditions, such as after ischemic insult (Guitart *et al*., 2016) and in neurodegenerative diseases (ND) (Vassileff *et al.*, 2020). A shared feature of ND (e.g., Alzheimer's disease (AD), Parkinson's disease (PD), and prion‐diseases (PrD)) is the aggregation and deposition of characteristic misfolded proteins in distinct areas of the brain, which propagate during the disease course (Hill, 2019; Rastogi *et al*., 2021). Numerous studies have shown that EVs carry and accumulate these pathological proteins (such as amyloid‐beta (Aβ) and tau in AD, alpha‐synuclein in PD, or transmissible prions in PrD), either inside or at the EV surface/corona, and mediate their propagation (Fevrier *et al*., 2004; Howitt *et al.*, 2021; Mattei *et al*., 2009; Perez‐Gonzalez *et al*., 2012; Ruan *et a* *l*., 2020). Given the capability of EVs to cross the blood‐brain‐barrier (BBB) (Ramos‐Zaldívar *et al*., 2022), there is a growing interest in defining their cargo in different biofluids, as brain‐derived EVs (BDEVs) may provide valuable information about the (patho)physiological state of the brain and serve as disease indicators and biomarkers (Vassileff *et al.*, 2020). However, despite their promising potential, further research is needed to understand the complete role of BDEVs in ND pathophysiology.

Given the growing interest in and the relevance of isolating EVs in a near‐to‐native state from complex tissues such as the brain, the number of protocols is increasing (Brenna *et al*., 2020; D'Acunzo *et al*., 2021; Gomes *et al*., 2023; Huang *et al.*, 2020; Huang, Arab *et al*., 2023; Hurwitz *et al*., 2018; Pait *et al*., 2024; Su *et al*., 2021; Vella *et al.*, 2017; Zhang *et al.*, 2023). Brain EV isolation is challenging since BDEVs need to be released from the extracellular matrix (ECM), which consists of an intricate meshwork of (glyco‐)proteins, large proteoglycans, and other molecules. With current EV isolation methods, different approaches, including enzymatic treatment and gentle mechanical dissociation, are employed to address this challenge (Brenna *et al.*, 2021). However, the commonly used enzymes like collagenase have already been shown that can alter the protein landscape by introducing unwanted cleavages of proteins present at the EV membrane, like the cellular prion protein (PrP^C^) (Brenna *et al.*, 2020), which has an important role in the pathophysiology of several ND (Laurén *et al*., 2009; Prusiner, 1982; Urrea *et al*., 2018) and may be relevant in BDEV uptake, trafficking, and function in brain pathologies (Brenna *et al.*, 2020; D'Arrigo *et al*., 2021; Falker *et al.*, 2016; Guitart *et al.*, 2016; Heisler *et al.*, 2018). This ‘artificial’ and unintended modification of proteins may be particularly relevant for EV membrane proteins and the EV corona content and may introduce artifacts that interfere with the study of EV biology, especially with regard to diagnostic (e.g., disease biomarker) and therapeutic (e.g., targeted drug‐delivery) potential (Tóth *et al.*, 2021; Wolf *et al.*, 2022).

The present study describes the effect of collagenase digestion used during isolation of BDEVs, which produces undesired protein cleavage of EV‐associated proteins in brain tissue homogenates and cell‐derived EVs. In order to avoid this effect, we explored the possibility of isolating BDEVs with either a reduced amount of collagenase or entirely excluding collagenase from the process. The BDEVs isolated with both approaches were characterized by nanoparticle tracking analysis (NTA), transmission electron microscopy (TEM), western blot (WB), multiplexed mRNA panels, and mass spectrometry, showing no major differences between protocols. However, even a minor collagenase treatment leads to pruning of some EV proteins. We show that the non‐enzymatically‐based method allows the preservation of membrane proteins while still yielding high quantities of BDEVs. This method provides more reliable BDEV samples for diagnosis and research. Intriguingly, our results also show the presence of the Golgi protein GM130 in non‐enzymatically isolated BDEVs, therefore questioning its use as a suitable negative marker for EV validation.

## 2 MATERIALS AND METHODS

### 2.1 Biological samples and respective ethics statements

#### 2.1.1 Human samples

The use of patient specimens (*n* = 6, cohort B) for research after completed diagnosis and upon anonymization was in accordance with local ethical standards and regulations at the University Medical Center Hamburg‐Eppendorf (UKE, Germany). Moreover, autopsy samples of the frontal cortex from non‐demented male controls (*n* = 9, cohort A) were donated from the brain bank of the Institute of Neuropathology, HUB‐ICO‐IDIBELL Biobank, *Hospitalet de Llobregat*, Barcelona, Spain, in compliance with the Spanish biomedical research regulations, including *Ley de la Investigación Biomédica* 2013 and *Real Decreto de Biobancos* 2014, and approval of the Ethics Committee of the Bellvitge University Hospital (HUB). Additional information, including age, post‐mortem intervals (PMI), neuropathological evaluation, and National Institute of Aging (NIA) staging, are shown in Table 1. BDEVs were isolated twice from the PO1, PO2, and PO3 samples for TEM, WB, NTA, proteomics, and nCounter^®^ panels. BDEVs isolated from the PO4, PO5, and PO6 samples were used for the NTA and nCounter^®^ panels. Brain homogenates from PO1, PO2, and PO3 samples were prepared for collagenase digestion and as loading controls for the WBs. BDEVs isolated from PO7, PO8, and PO9 were used for WB. BDEVs were isolated from the PO10, PO11, PO12, PO13, PO14, and PO15 samples for TEM, WB, and NTA.

**TABLE 1:** Frontal cortex autopsy tissues used for different BDEV preparations with collagenase‐assisted and collagenase‐free methods.

N°	IDs	Cohort	Sex	Age	PMI	NIA scoring	Neuropathological findings
1	**PO1**	A	M	78	2 h 15 min	A0, B0, C0	Lacunar infarcts
2	**PO2**	A	M	67	5 h	A0, B0, C0	Lacunar infarcts
3	**PO3**	A	M	71	12 h	A0, B0, C0	No neuropathological findings
4	**PO4**	A	M	61	3 h 55 min	A0, B0, C0	Metabolic encephalopathy
5	**PO5**	A	M	85	5 h 45 min	A0, B0, C0	Lacunar infarcts in striatum
6	**PO6**	A	M	64	3 h 30 min	A0, B0, C0	Mesial sclerosis
7	**PO7**	A	F	75	3 h	A0, B0, C0	Lacunar infarcts
8	**PO8**	A	F	74	2 h 45 min	A1, B1, C0	Amyloid and Tau pathology
9	**PO9**	A	F	75	4 h 55 min	A1, B1, C0	Amyloid and Tau pathology
10	**PO10**	B	F	66	3 days	A0, B0, C0	No neuropathological findings
11	**PO11**	B	F	79	4 days	A0, B0, C0	No neuropathological findings
12	**PO12**	B	F	59	>2 days	A0, B0, C0	Inflammatory profile (CD20+)
13	**PO13**	B	F	69	>2 days	A0, B0, C0	Inflammatory profile (CD8+)
14	**PO14**	B	F	68	2 days	A0, B0, C0	Encephalitis and CD8+
15	**PO15**	B	F	55	2 days	A0, B0, C0	Leukoencephalopathy

#### 2.1.2 Mice

In this study, we performed no experiments with living animals. Breeding and the euthanasia procedure to obtain brain tissue were approved by the ethical research committees of respective national/local authorities: *Freie und Hansestadt Hamburg, Behörde für Gesundheit und Verbraucherschutz*, Hamburg, Germany (ORG1023). Adult male and female C57BL/6J mice (*n* ♀ = 9, *n* ♂ = 6; 35–53 weeks old) were purchased from Charles River Laboratories (Paris, France). Sampling was performed following the protocols and guidelines of the Ethical Committee and the directives of the European Union Council 86/609 and 2010/63.

#### 2.1.3 Cell lines

Neuro‐2a cells (N2a) were ordered via DSMZ (ACC 148) in the Institute of Neuropathology, UKE, Hamburg (ATCC: CCL‐131). Cells were maintained in DMEM (Gibco) supplemented with 10% FBS (Invitrogen) at 37°C and 5% CO_2_ atmosphere.

### 2.2 Homogenization and collagenase digestion of total brain tissue

About 20% (w/v) homogenates (200 mg/1 mL) of C57BL/6J mouse forebrain or human frontal cortex were prepared in PBS with a manual dounce homogenizer. About 100 µL of homogenate were treated with collagenase D (Roche, LOT:70507825: 0.15 U/mg) (i) high concentration (2 mg/mL); (ii) collagenase D low concentration (0.5 mg/mL); and collagenase type III (Worthington, LOT: F8P18802: 315 U/mg) (iii) high concentration (1.5 mg/mL) and (iv) collagenase type III low concentration (0.38 mg/mL). A control homogenized with PBS and freshly added protease inhibitor (PI) cocktail (complete EDTA‐free tablet, Roche) was included to assess the role of endogenous proteases. One tube was left without enzymes as a control (without PI). Samples were then incubated for 30 min at 37°C in a thermomixer at 500 rpm. The reaction was stopped by adding 200 µL of a stop solution of 2× RIPA buffer (100 mM Tris Base, 300 mM NaCl, 2% NP40, 1% Na‐Deoxycholate and 0.2% SDS at pH = 8) with 0.1 M EDTA and freshly added protease inhibitors (PI). Tubes were vortexed and left on ice for 10 min to ensure the activity of inhibitors and detergent, then centrifuged at 12,000 × *g* for 10 min at 4°C. The supernatant was collected, 4× sample buffer (SB, 240 mM Tris Base, 8% SDS, 40% glycerol, and 0.2% bromophenol blue at pH = 6.8) containing 5% β‐mercaptoethanol (to a final concentration of 1×) was added, and the samples were boiled at 95°C for 10 min. The remaining sample was stored at −80°C for further western blotting analysis (see below).

### 2.3 Isolation of cell culture supernatant EVs

**FIGURE 1:**
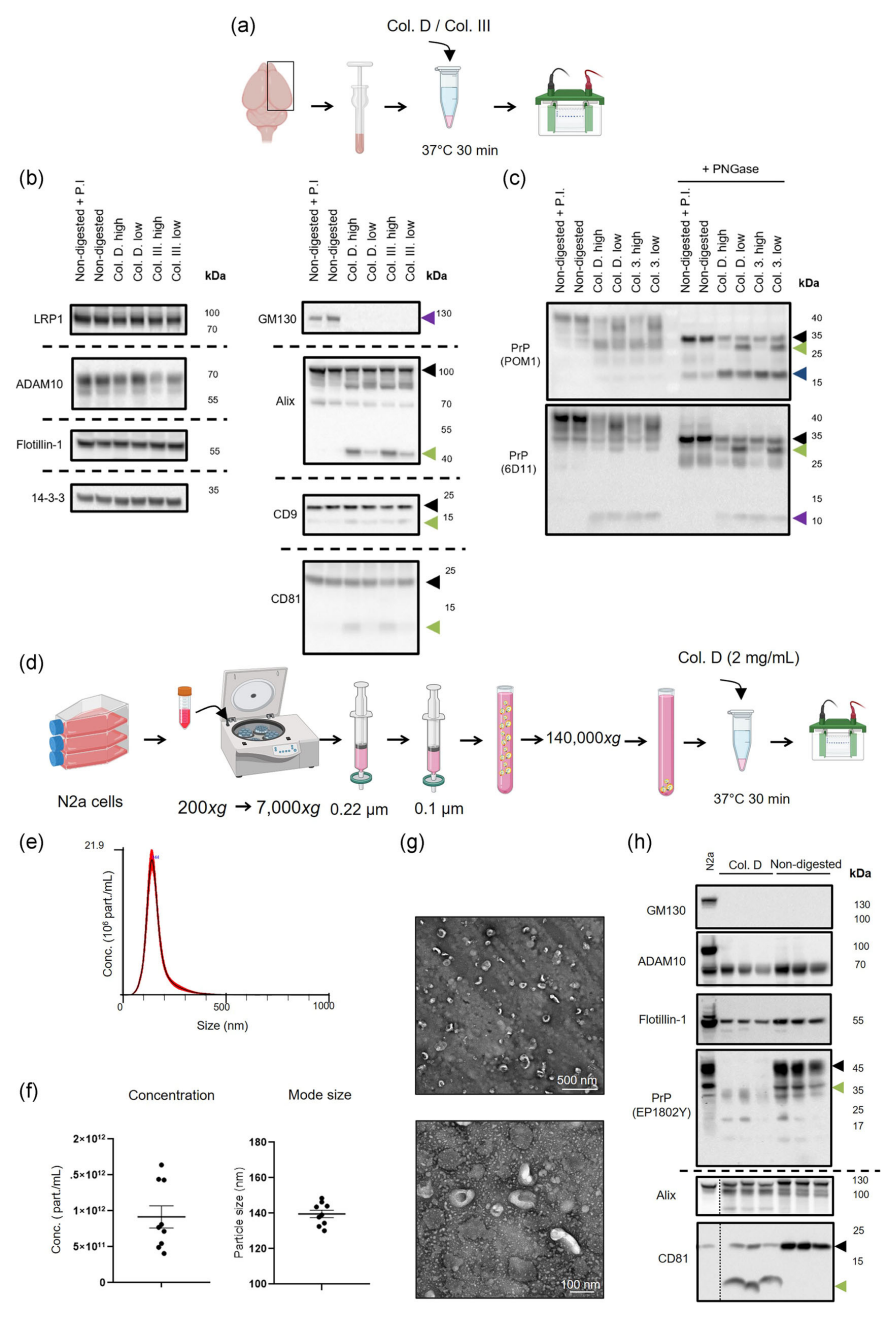
Collagenase digestion induces undesired cleavage of EV‐relevant (surface) proteins in both brain tissue and cell‐derived EVs. (a) Schematic drawing depicting the collagenase treatment in brain tissue. One mouse hemisphere was dissected and homogenized manually in PBS. Subsequently, 100 µL of homogenate underwent treatment with collagenase D at either high concentration equivalent to 2 mg/mL or low concentration 0.5 mg/mL and collagenase III at either high (1.5 mg/mL) or low (0.4 mg/mL) concentrations and was then incubated for 30 min at 37°C before being subject to WB analysis. (b) Representative WBs of relevant proteins used as EV markers after collagenase treatment. In the left panel, LRP1, Flotillin‐1, and 14‐3‐3 remained unaffected by both collagenase treatments, but ADAM10 amounts were diminished with collagenase III. However, in the right panel, Alix, CD9, and CD81 are visibly cut, producing lower molecular weight (MW) fragments (indicated by green arrowheads) in addition to the expected MW (black arrowheads). GM130 (purple arrowhead) was also found to be digested by collagenase treatments. Non‐digested samples with (+) and without protease inhibitors (PI) were used as controls. (c) PrP^C^ patterns after collagenase treatment and with or without PNGase F digestion were detected by POM1 and 6D11 antibodies. The *N*‐glycan removal shows exacerbated PrP‐C1‐like (∼15 kDa; blue arrowhead) and PrP‐N1‐like (∼10 kDa; purple arrowhead) fragments, and another generated fragment (green arrowheads) slightly shorter than full‐length PrP^C^ (black arrowheads). (d) Schematic drawing depicting the isolation and collagenase treatment of N2a‐EVs. EVs were isolated by differential centrifugation and filtration (*n* = 9). Then, samples were divided into two tubes, each containing 3.0×10^6^ EVs, and treated or not with collagenase D (2 mg/mL; 30 min incubation at 37°C) followed by WB analysis. (e) Representative size distribution graph from NTA before collagenase treatment. (f) The concentration of particles per mL and mode size analysis (in nm) of N2a‐EVs were measured with NTA (*n* = 9). Data are presented as mean ± S.E.M. (g) Representative TEM images of N2a cell‐derived EVs. (h) Representative WBs of N2a‐EVs (*n* = 3 each) treated or not with collagenase D. N2a cell lysate was used as a loading control. The outer‐membrane proteins CD81 and PrP^C^ are altered, but not ADAM10 and the luminal markers Flotillin‐1 and Alix. Schema (a) created in BioRender. Matamoros, A. (2024) BioRender.com/a98v729 and schema (d) created in BioRender. Matamoros, A. (2024) BioRender.com/k55o413.

Cells were cultured to approximately 80% confluency in T175 cell culture flasks in DMEM (Gibco) with 10% FBS (Gibco). About 18–24 h before EV isolation, cells were washed with PBS, and the medium was changed to the same DMEM but supplemented with 10% exosome‐depleted FBS (EXO‐FBS‐50A‐1, System Bioscience). The media from three flasks (24 mL each) was collected on the day of isolation, and differential centrifugation was performed to remove cell debris from the culture medium (200 × *g* for 5 min and 7,000 × *g* for 20 min). The whole procedure was conducted on ice, and centrifugations were performed at 4°C to avoid degradation. Subsequently, the supernatant was passed through a 0.22 µm filter using the SteriFlip filter system (Merck) and a PVDF 0.1 µm filter (Merck). The flow‐through was then transferred into 12 mL polypropylene tubes (Beckman Coulter) and centrifuged at 140,000 × *g* (Beckman Coulter Optima L100 XP, SW40Ti, Swing bucket rotor) for 70 min. The final pellet was resuspended in 90 µL PBS containing PI. See Figure 1d for a scheme of the isolation process.

### 2.4 Isolation of brain‐derived EVs

All brain tissues used here were previously stored at −80°C. During all procedures, protein low‐binding tubes (Eppendorf) were used. PBS used in all EV‐related experiments was prefiltered through a 0.1 µm filter to avoid impurities. For all the EV resuspensions, a protease inhibitor cocktail at 1× was freshly added. BDEVs were isolated from mouse brain tissues (C57BL/6J, 35–53 weeks old, female or male) or human brain tissues (see human samples section for details) as follows. The protocol used is based on the one published by Crescitelli *et al*. (2021), where we added some minor modifications to adapt it to our equipment, reduce the protease activity (i.e., by including PI in several steps and excluding or decreasing the amount of collagenase D). The adapted protocol was as follows.

The tissue dissection and the rest of the procedure were performed on ice to protect samples from degradation. About 100–200 mg of frozen brain tissue was minced with a scalpel in a glass petri dish with cold RPMI media (Gibco). 0.55 mL of media was added to each 100 mg of tissue to normalize the media/tissue ratio in all the samples. The mixture was transferred into a 1.5 mL tube (with a cut 1 mL tip), and DNAse I (Roche) was added to a final concentration of 40 U/mL. Collagenase D at low concentration (0.5 mg/mL equivalent to 0.075 U/mL) or no collagenase (w/o) was added to the samples. Tubes were incubated in a thermomixer at 37°C for 20 min at 500 rpm, and the mixture was pipetted up and down 15 times every 5 min. To stop the endogenous protease activity, RPMI media with 10× concentrated PI (1/10 of the initial volume) was added to the tubes. Subsequently, samples were centrifuged at 300 × *g* (5 min), 2,000 × *g* (10 min), and 10,000 × *g* (10 min). The 10,000 × *g* pellet containing large BDEVs was not further included in the isolation process to avoid larger vesicles and debris and kept as a “10K control”. Afterward, the supernatant was transferred into a 5 mL polypropylene tube (Beckman Coulter), PBS was added to reach a 4.5 mL tube filling, and samples were centrifuged at 120,000 × *g* for 100 min at 4°C in the L‐60 Optima Ultracentrifuge (Beckman Coulter, SW55 TI rotor, Swing bucket rotor). Then, the supernatant was discarded, and the pellet was resuspended with 300 µL PBS (+PI). About 240 µL of resuspended BDEVs were mixed with 60% OptiPrep at the bottom of the same tube, while 60 µL were stored at −80°C as the “pre‐gradient control” for WB analysis. To set up the gradient, 1.3 mL of 30% and 10% OptiPrep (Stemcell) densities (prepared as described in Crescitelli *et al*., 2021) were layered on top. Finally, 400 µL of PBS were added on top, and the gradient was centrifuged at 185,500 × *g* for 120 min at 4°C. Four fractions were collected (F1: 1 mL, F2: 1.3 mL, F3: 0.65 mL, F4: 1.3 mL) into 1.5 mL tubes. BDEVs were expected to mainly concentrate between the 10% and 30% interface. Fractions were then further diluted with PBS, stored at 4°C overnight, and centrifuged at 120,000 × *g* for 80 min at 4°C the next day to pellet the EVs and wash out the OptiPrep. After carefully removing the supernatant, BDEVs were resuspended gently with 60–90 µL of PBS (+PI). Samples were transferred to 1.5 mL tubes, stored at 4°C (if downstream NTA analysis was performed within 1 week), prepared for further biochemical and imaging analysis (see below), or frozen directly at −80°C.

### 2.5 Nanoparticle tracking analysis (NTA)

Samples were diluted in PBS depending on estimated EV concentrations. Cell culture‐derived EVs were diluted with PBS 1:500 or 1:1,000, while tissue‐derived samples were diluted 1:125, 1:250, or 1:500. Before dilution, human BDEVs were treated with 4% paraformaldehyde (PFA) for biosafety reasons. Five videos of 30 s duration were recorded (settings: camera level = 16, screen gain = 2) with the NanoSight microscope (LM14, Malvern). The' Process' function of NanoSight NTA 3.0 software was used to analyse the recordings (settings: detection threshold = 6 and screen gain = 10). PBS (with or without PFA traces mimicking the final buffer concentration in the BDEV samples) was also analysed as background control and showed almost no signal.

### 2.6 Negative staining and transmission electron microscopy (TEM)

About 5 µL of BDEVs were fixed with 16% PFA to a final concentration of 4%. Five µL of the fixed sample were then added to a formvar/carbon‐coated 200 mesh copper grid (#ECF200‐Cu‐50, Science Services) and left for 20 min in a dry environment. Thereafter, samples were transferred to drops of PBS (three times for 2 min each). The samples were further stained with 2% methylcellulose‐uranyl acetate on ice and looped out on a filter paper after 10 min. EVs were analysed and imaged with a transmission electron microscope (Jeol JEM2100Plus) equipped with a XAROSA CMOS camera (Emsis).

### 2.7 Collagenase treatment of cell‐derived EVs

About 3 × 10^10^ particles of N2a‐EVs (measured by NTA) were either incubated with or without 2 mg/mL collagenase D at 37°C for 20 min in a thermomixer at 500 rpm. The reaction was stopped by adding 1/8 of RIPA 8× buffer supplemented with an 8× PI to reach a 1× concentration. Samples were left on ice for 10 min for EV lysis and prepared for WB analyses by adding 4× SB (with 5% β‐mercaptoethanol) followed by boiling at 95°C for 10 min.

### 2.8 Protein quantification

BCA Assay Kit (Pierce) was used to quantify the total protein content in brain homogenates, and the microBCA Assay Kit (Pierce) was used to quantify the BDEV samples, both according to their manuals. In both cases, the extinction of the bicinchoninic acid (BCA) was measured at 562 nm utilizing the µQuant spectrometer (BioTek). A standard curve was set up using albumin to determine the protein concentration of each sample.

### 2.9 Protein deglycosylation

Deglycosylation of total brain homogenates was performed according to the PNGase F kit instruction manual (#P0704S, New England Biolabs). About 40 µg of protein were digested with 1,000 U of PNGase F enzyme.

### 2.10 Sample preparation for SDS‐PAGE

Brain lysates from mouse or human tissues were prepared as a 10% homogenate with RIPA buffer (50 mM Tris Base, 150 mM NaCl, 1% NP40, 0.5% Na‐Deoxycholate and 0.1% SDS at pH = 8) with freshly added PI for being used as brain homogenate (BH) controls. N2a cell lysates were prepared using the same buffer. After brain or cell lysis for 10 min on ice, protein lysates were obtained in the supernatants after a 12,000 ×*g* centrifugation for 10 min. EV samples (in PBS, see below) were prepared by adding 8× RIPA buffer (400 mM Tris Base, 1.2 M NaCl, 8% NP40, 4% Na‐Deoxycholate and 0.8% SDS at pH = 8) to a final concentration of 1× to not excessively increase the sample volume. Gentle pipetting and a 10 min incubation were done to guarantee EV lysis. Finally, in all the cases, 4× SB (containing 5% β‐mercaptoethanol) to a final concentration of 1×) was added, and the samples were boiled at 95°C for 10 min.

### 2.11 SDS‐PAGE and western blot analysis

BDEV from 25–30 mg of original tissue were loaded per lane and cell‐derived EVs by the particle number (measured by NTA). Normalized samples were loaded on pre‐casted 4%–12% Bis‐Tris protein gels (Invitrogen) with a suitable molecular weight marker (10–180 kDa) and loading controls, that is, either BH or cell lysate. After electrophoretic separation in MES/SDS buffer (0.5 M MES, 0,5 M Tris base, 1% SDS, and 9.6 mM EDTA), proteins were transferred by wet‐blotting with Tris‐Glycine buffer (250 mM Tris base, 1.92 M Glycine and 10% methanol) onto a 0.2 µm pore‐sized nitrocellulose membrane (BioRad). After completion of the transfer, the total amount of protein was detected using the Revert™ 700 Total Protein Stain Kit (LI‐COR) and the Odyssey DLx imaging system (LI‐COR) according to the manufacturer's manual. Subsequently, membranes were blocked for 1 h at room temperature (RT) with 5% non‐fat dry milk diluted in TBS‐T buffer (100 mM Tris base, 1.4 M NaCl, and 1% Tween‐20 at pH = 7.4). Membranes were incubated overnight with primary antibodies at 4°C on a shaking platform. The following primary antibodies in a dilution of 1:1,000 were used: anti‐14‐3‐3 (#9636S, Cell Signalling), ADAM10 (#ab124695, Abcam), anti‐Alix (#92880S, Cell Signalling), anti‐CD81 human‐specific (D3N2D) (#56039S, Cell Signalling) and mouse‐specific (D502Q) (#10037S, Cell Signalling), anti‐CD9 (#13403S, Cell Signalling, or #ab92726, Abcam), anti‐Flotillin‐1 (#610820, BD Biosciences), anti‐Flotillin‐2 (#ab96507, Abcam), anti‐GM130 (#610822, BD Biosciences, or #ab52649, Abcam), anti‐Laminin A/C (#SAB4200236, Merck, or #ab108595, Abcam), anti‐LRP‐1 (#ab92544, Abcam), anti‐PrP^C^ clone POM1 (#MABN2285, Merck), anti‐PrP^C^ clone EP1802Y (#ab52604, Abcam), anti‐PrP^C^ clone 6D11 (#808002, BioLegend) and anti‐PrP^C^ clone 3F4 (#MAB1562, Merck). The next day, corresponding HRP‐conjugated secondary antibodies (Anti‐Rabbit, #W4011 and Anti‐Mouse #W4021, both from Promega) were incubated (1:4,000) for 1 h at RT in 5% non‐fat dry milk (in TBS‐T buffer). Detection was performed with Pierce ECL Pico or Femto substrate (Thermo Fisher Scientific) using the Chemidoc XRS+ imaging system (BioRad).

### 2.12 Protein extraction and tryptic digestion of EVs for mass spectrometry analysis

EV samples were diluted 1:1 in 100 mM triethyl ammonium bicarbonate (TEAB), and 1% w/v sodium deoxycholate (NaDoC) and dissolved to a concentration of 70% Acetonitrile (ACN). Two µg carboxylate modified magnetic beads (GE Healthcare Sera‐Mag™) at 1:1 (hydrophilic/hydrophobic) in methanol were added following the SP3‐protocol workflow (Hughes *et al.*, 2019). Samples were shaken at 1,400 rpm for 18 min at RT and placed on a magnetic rack. The supernatant was removed, and magnetic beads were washed two times with 100% ACN and two times with 70% Ethanol. After resuspension in 50 mM ammonium bicarbonate, disulfide bonds were reduced in 10 mM dithiothreitol for 30 min, alkylated in the presence of 20 mM iodoacetamide for 30 min in the dark, and digested with trypsin (sequencing grade, Promega) at 1:100 (enzyme: protein ratio) at 37°C overnight while shaking at 1,400 rpm. Beads were dissolved in 95% ACN and shaken at 1,400 rpm for 10 min at RT to bind tryptic peptides to the beads. On the magnetic rack, the supernatant was removed, and beads were washed two times with 100% ACN. Elution was performed with 2% DMSO in 1% formic acid. The supernatant was dried in a vacuum centrifuge and stored at ‐20°C until further use.

### 2.13 Peptide library generation with high pH fractionation

Three human (PO1, PO2, and PO3) and three mouse brain homogenates were used for library generation. Protein extraction was performed with 7 M urea, 2 M Thiourea, 4% CHAPS, 150 mM DTT, and 0.5% Ampholyte. Lysates were concentrated to a final concentration of 4 mg/mL. For each sample, 50 µg of protein was subjected to tryptic digestion, following the SP3 protocol (Hughes *et al.*, 2019). Digests from different samples were combined before fractionation. In total, 50 µg of tryptic peptides were used for High pH RP‐HPLC using a 25 cm ProSwift™ RP‐4H capillary monolithic column (Thermo Scientific) on an Agilent 1200 series HPLC (high‐pressure liquid chromatography) system. A gradient was applied for a total of 45 min with a flow rate of 0.2 mL/min starting at 96.7% eluent A (10 mM NH_4_HCO_3_) and 3.3% eluent B (10 mM NH_4_HCO_3_ in 90% ACN) for 5 min, rising to 38.5% B in 20 min and increasing to 95.0% in 1 min for 10 min and re‐equilibrated to 3.3% B for 8 min. Thirty fractions were collected on an Äkta Prime Plus fraction collector, pooled into 13 peptide library fractions, and dried in the vacuum centrifuge.

### 2.14 Liquid–chromatography‐coupled tandem mass spectrometry (LC–MS/MS)

Prior to LC–MS/MS analysis, EV samples and peptide library fractions were dissolved in 0.1% FA to a final concentration of 1 µg/µL. For LC–MS/MS measurements, 1 µg tryptic peptides were injected. Measurements of EV samples were performed on an orbitrap MS (QExactive, Thermo Fisher) coupled to a nano‐UPLC (Dionex Ultimate 3000 ultra‐performance liquid chromatography system, Thermo Fisher Scientific). Measurements of peptide library fractions were performed on a quadrupole‐ion‐trap‐orbitrap MS (Orbitrap Fusion, Thermo Fisher Scientific) in orbitrap‐orbitrap configuration. Chromatographic separation of peptides was achieved with a two‐buffer system (buffer A: 0.1% FA in ultrapure H_2_O, buffer B: 0.1% FA in ACN). Attached to the UPLC was a peptide trap (100 µm × 200 mm, 100 Å pore size, 5 µm particle size, C18, Thermo Fisher Scientific) for online desalting and purification followed by a 25 cm C18 reversed‐phase column (75 µm × 250 mm, 130 Å pore size, 1.7 µm particle size, Peptide BEH C18, Waters). Peptides were separated using an 80‐min gradient with linearly increasing ACN concentration from 2% to 30% ACN in 65 min. Eluting peptides were ionized using a nano‐electrospray ionization source (nano‐ESI) with a spray voltage of 1,800 V, transferred into the MS, and analysed in data‐dependent acquisition (DDA) mode. For each MS1 scan, ions were accumulated for a maximum of 240 ms or until a charge density of 1 × 10^6^ ions (AGC Target) was reached. Fourier‐transformation‐based mass analysis of the data from the orbitrap mass analyser was performed, covering a mass range of 400–1,200 *m*/*z* with a resolution of 70,000 (at *m*/*z* = 200) for EV samples on the orbitrap MS and a resolution of 60,000 for peptide library on the quadrupole‐ion‐trap‐orbitrap MS in orbitrap‐orbitrap configuration. Peptides with charge states between 2+ and 5+ above an intensity threshold of 1 × 10^5^ were isolated within a 2 m/z isolation window from each precursor scan and fragmented with a normalized collision energy of 25% using higher energy collisional dissociation (HCD). MS2 scanning was performed at a resolution of 15,000 for EV samples on the orbitrap MS and a resolution of 17,500 for peptide library on the quadrupole‐ion‐trap‐orbitrap MS in orbitrap‐orbitrap configuration, covering a mass range from 100 *m*/*z* and accumulated for 50 ms or to an AGC target of 1 × 10^5^. Already fragmented peptides were excluded for 15 s.

### 2.15 Raw data processing

LC–MS/MS from DDA were searched with the Sequest algorithm integrated into the Proteome Discoverer software (Version 2.4.1.15, Thermo Fisher Scientific) against a reviewed mouse database obtained in October 2020, containing 17,053 entries and a reviewed human database, obtained in June 2021, containing 20,386 entries. Carbamidomethylation was set as a fixed modification for cysteine residues, and the oxidation of methionine and pyro‐glutamate formation at glutamine residues at the peptide N‐terminus, as well as acetylation of the protein N‐terminus, were allowed as variable modifications. A maximum number of two missing tryptic cleavages was set. Peptides between 6 and 144 amino acids were considered. A strict cutoff (FDR < 0.01) was set for peptide and protein identification. LC–MS/MS from peptide library fractions were handled in a separate processing step within the software. A multi‐consensus workflow was applied to sample and library files (.mgf) to generate a combined output and increase the protein identification rate for individual samples through the feature mapper. Normalization was performed in specific protein amount mode based on the EV marker proteins FLOT1, FLOT2, CD9, CD63, CD37, CD81, CD82, CD151, PDCD6IP, TSG101, RAB1A, RAB1B, RAB2A, RAB2B, RAB3A, RAB3B, RAB4A, RAB4B, RAB5A, RAB5B, RAB6A, RAB6B, RAB7A, RAB7B, RAB8A, RAB8B, RAB9A, RAB9B, RAB10, RAB11B, ANXA1, ANXA2, ANXA3, ANXA4, ANXA5, ANXA6, ANXA7, ANXA11. Protein abundances for individual samples were exported and submitted to subsequent statistical analysis. Protein abundances for library fractions were discarded. Data is available via ProteomeXchange with the identifier PXD045737.

### 2.16 mRNA content analysis with nCounter^®^ panels (Nanostring)

Samples were processed in the panel without prior RNA isolation, as published previously (Bub *et al.*, 2022). Briefly, BDEVs were isolated from human brain tissues, as explained before (with and without collagenase), and fractions F1 and F2 of the same sample were pooled. PBS was added to the pooled samples and pelleted again for 2 h at 120,000 × *g*. After discarding the PBS completely, the BDEVs were resuspended in 10 µL of RTL Lysis Buffer (Qiagen) diluted 1:3 in RNAse‐free H_2_O. Five µL were loaded from each sample in the NanoString nCounter^®^ Neuropathology panel (#XT‐CSO‐HNROP1‐12, NanoString Technologies).

### 2.17 Statistical and bioinformatics analyses

Statistical analysis was performed with GraphPad PRISM 8 (GraphPad Software, USA) or R. Unless otherwise stated, data are plotted as the mean ± SEM or ±SD. The normality of the distributions in the NTA‐derived results was checked using the Shapiro–Wilk test. If all the samples passed the normality test, the Ordinary one‐way ANOVA test and Bonferroni's multiple comparisons test were performed to validate the statistical differences; if not, the non‐parametrical Kruskal–Wallis test was used.

For the statistical and bioinformatics analyses of the proteomics data sets, we mainly relied on Perseus software (Max Planck Institute of Biochemistry, Martinsried, Germany) and various packages in R. Proteins which had at least two valid values in at least one of the groups were kept for further analysis. Initially, proteins with missing values were excluded from the Principal component analyses and heatmaps. Each sample was then normalized by the median protein area per sample, and paired comparisons using Student's *t*‐test were performed using Perseus. Proteins were considered to be significantly different in abundance if the Student's *t*‐test‐based *p*‐value was ≤0.05 and at least a 1.5‐fold change in either direction was observed. Proteins were considered uniquely present in a certain group when they were expressed in at least two out of three replicates for that particular group and were simultaneously absent in the other group during pairwise comparison.

Principal component analysis (PCA) was carried out using the *prcomp* function of the base R. Resultant PCA plots were composed utilizing the ggplot2 (version 3.3.3, 2016). Hierarchical clustering and heatmaps were prepared using *heatmp.2* function in the gplot package (version 3.1.1, 2021). Correlation plots were prepared using ggcorrplot package (version 0.1.4, 2021). Peason's coefficient values were obtained using base R.

Gene ontology (GO) analyses were performed utilizing the clusterProfiler, enrichR, org.Hs.eg.db, org.Mm.eg.db, and topGO packages. Overrepresentation analysis (ORA) was performed to find associations of differentially upregulated proteins along with proteins uniquely detected in F1 (+ and −) and F2 (+ and −) individually with the GO category ‘Cellular Components’ with the following analysis parameters: *p*‐valueCutoff = 0.05, *q*‐valueCutoff = 0.2, minGSSize = 5, and maxGSSize = 500.

Proteins constituting the EV preparations were checked for their presence in the Vesiclepedia database (Kalra *et al.*, 2012; Pathan *et al.*, 2019; Chitti *et al.*, 2024), using FunRich enrichment software (Pathan *et al.*, 2015) on 10th December 2023. Mouse proteins were compared to the human Vesiclepedia database, as the corresponding mouse database was not supported on the FunRich platform.

For the nCounter^®^ panel analysis, all initial statistical work‐up was performed using nSolver analysis software (version 4.0.70). For the downstream analyses of mRNA expression profiles, genes with raw expression scores not exceeding the average plus two standard deviations of all corresponding negative control probes were removed from the analysis. All expression values below the 20 reads threshold were imputed to 20. Normalization was based on the tetraspanin mRNAs present on the panel, that is, *CD14*, *CD33*, *CD34*, *CD4*, *CD40*, *CD44*, *CD68*, *CD8A*, and *CD9*. All genes with no detection in any of the samples were excluded from the analysis. An mRNA was considered differentially expressed if the corresponding absolute log2‐fold change (log2FC) was ≥0.584 and the *p*‐value ≤0.05. PCA, heatmaps, and correlation plots were prepared as those for proteomics, as described above.

## 3 RESULTS

### 3.1 Collagenase treatment of brain homogenates impact on key BDEV proteins, including PrP^C^

In a previous study, our groups reported the enrichment of the proteolytically truncated PrP^C^‐C1 fragment (generated by physiological α‐cleavage Linsenmeier *et al.*, 2017) in BDEVs and described a certain proteolytic impact of the enzymatic tissue digestion (employed during BDEV isolation) on the PrP^C^‐C1/full‐length PrP^C^ ratio (Brenna *et al.*, 2020). In order to further examine this effect, we first digested mouse brain tissue with collagenase D and collagenase III, (both widely used in EV isolation protocols from tissue (e.g., Crescitelli *et al.*, 2021 and Su *et al.*, 2021)) (Figure 1a). Tissue was homogenised in PBS, and both low and high doses of collagenase D (high dose = 2 mg/mL, low dose = 0.5 mg/mL) and collagenase III (high dose = 1.5 mg/mL, low dose = 0.4 mg/mL) were used. As a control, we included non‐digested homogenate with and without the addition of protease inhibitors to exclude endogenous protease activity. The results indicated that the enzymes cleaved the EV‐related proteins Alix, CD9 and CD81, while 14‐3‐3, ADAM10, LRP1, and Flotillin‐1 remained unaffected (Figure 1b). Interestingly, GM130 clearly disappeared after all the enzymatic treatments, highlighting its sensitivity to collagenase digestion. We also conducted the same experiment using human brain tissue, which also demonstrated protein pattern modifications in CD81 and Alix but not CD9, suggesting different effects of the collagenases depending on the protein sequence variability among species (Figure S1a). We then specifically examined the effect of collagenase on PrP^C^ cleavage due to the relevance of different PrP^C^ fragments in its physiological functions (reviewed in Linsenmeier *et al.*, 2017; Mohammadi *et al.*, 2023). After collagenase digestion, the homogenate was treated with PNGase F to remove the *N*‐glycans and enable more clear distinctions in PrP^C^ patterns. Western blotting analyses using two anti‐PrP^C^ antibodies revealed two cleavages: one strikingly comparable (if not identical) with the physiological α‐cleavage (producing a C‐terminal fragment of ∼15 kDa and an N‐terminal one of ∼10 kDa) and another one artificially generated by collagenase in the N‐terminal region (Figure 1c). A similar protein digestion pattern was observed when human brain homogenate was tested (Figure S1b). Uncropped blots and corresponding total protein staining are included in Figure S2a,b.

### 3.2 Unlocking collagenase's impact on EVs: EV membrane proteins are cleaved, whereas intraluminal ones are spared

To discard the possibility of artifact generation resulting from EVs isolation from a complex system such as brain tissue, we examined the effect of collagenase D on isolated EVs from cultured N2a cells (N2a‐EVs). The N2a‐EVs were isolated by filtration and ultracentrifugation (Figure 1d). NTA analysis showed the usual size distribution with a concentration of 9.17 × 10^11^ ± 1.54 × 10^11^ particles per mL with a mode size of 139.6 ± 2.13 nm (Figure 1e,f). The typical cup shape of N2a‐EVs was confirmed using TEM (Figure 1g).

N2a‐EVs (3 × 10^10^) were incubated with collagenase D (2 mg/mL) for 30 min, and the resulting samples were subjected to WB analyses. CD81 and PrP^C^ were found to be clearly cleaved, generating ‘artificial’ protein fragments, whereas the EV‐luminal proteins Alix and Flotillin‐1 remained much less affected (Figure 1h). These proteins, along with the mature form of ADAM10, just showed slightly lower levels due to either the disruption of EVs during isolation and collagenase digestion and/or the digestion continuing after the EV lysis for western blotting. In line with this, we have previously observed that even a high concentration of protease inhibitors cannot effectively prevent collagenase activity (unpublished data). The Golgi protein GM130 was absent in all N2a‐EV samples (Figure 1h). For uncropped blots and corresponding total protein staining, see Figure S2c.

These results highlight the effect of collagenase on unspecific trimming and degradation of EV‐associated and ND‐related proteins, which could drastically affect downstream analyses and functionality of isolated BDEVs.

### 3.3 Characterization of a simplified and non‐enzymatic BDEV isolation protocol

**FIGURE 2:**
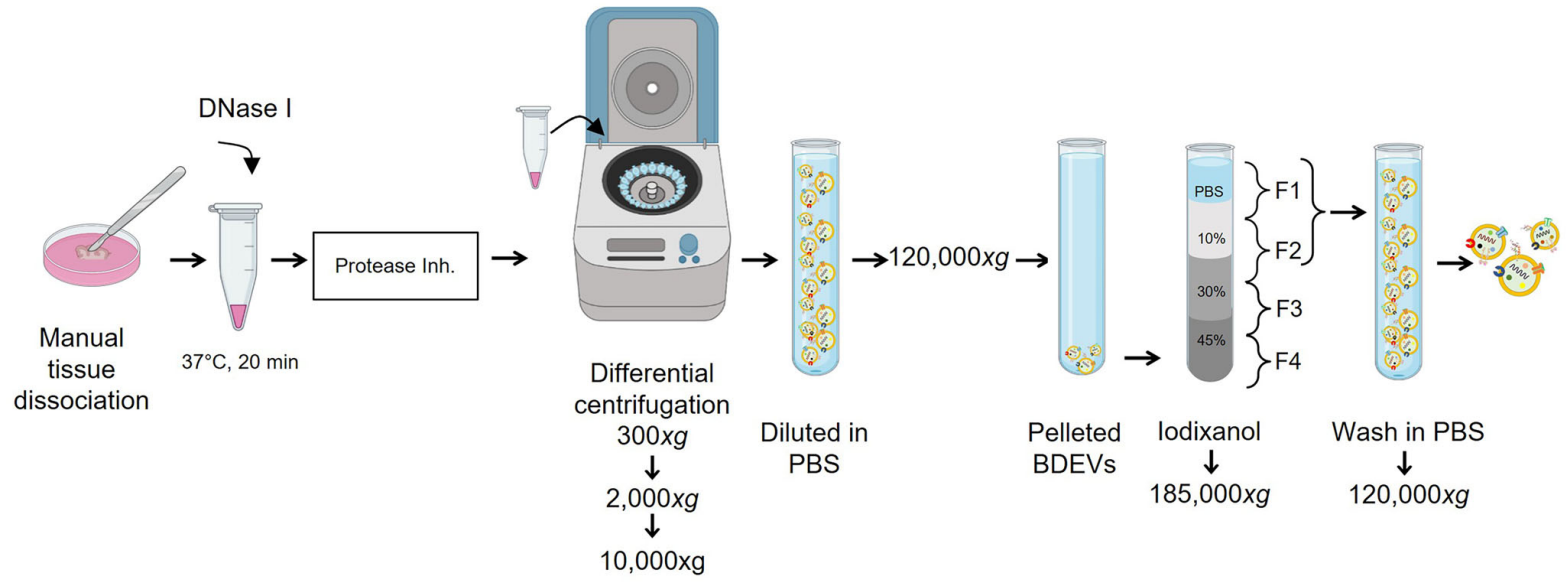
Simplified non‐enzymatic BDEV isolation protocol. Schematic representation of the new enzyme‐free BDEVs isolation protocol: human or murine brain tissue was manually minced in RPMI media and transferred to a new tube, where a DNAse I treatment was carried out at 37°C for 30 min. The reaction was stopped by adding a protease inhibitor cocktail. Next, differential centrifugation was applied (200 × *g*, 2,000 × *g*, and 10,000 × *g*), and the BDEV‐containing supernatant was diluted in PBS. BDEVs were then pelleted and washed at 120,000 × *g* before being separated in an iodixanol‐based gradient at 185,000 × *g*. Fractions (F) were collected, and F1 and F2 (which mainly contained the BDEVs) were washed in PBS and pelleted at 120,000 × *g*. Figure created in BioRender. Matamoros, A. (2024) BioRender.com/t17h786.

In order to mitigate the protein cleavage effect of collagenase digestion, we tested the possibility of isolating BDEVs with a reduced amount of collagenase D (0.5 mg/mL) and without any protease. Moreover, to reduce the protease activity, we reduced the enzymatic digestion time and included the addition of PI. We performed the BDEV isolation by slightly modifying a previously published protocol to isolate EVs from tumour tissues (Crescitelli *et al.*, 2021), as schematically shown in Figure 2.

**FIGURE 3:**
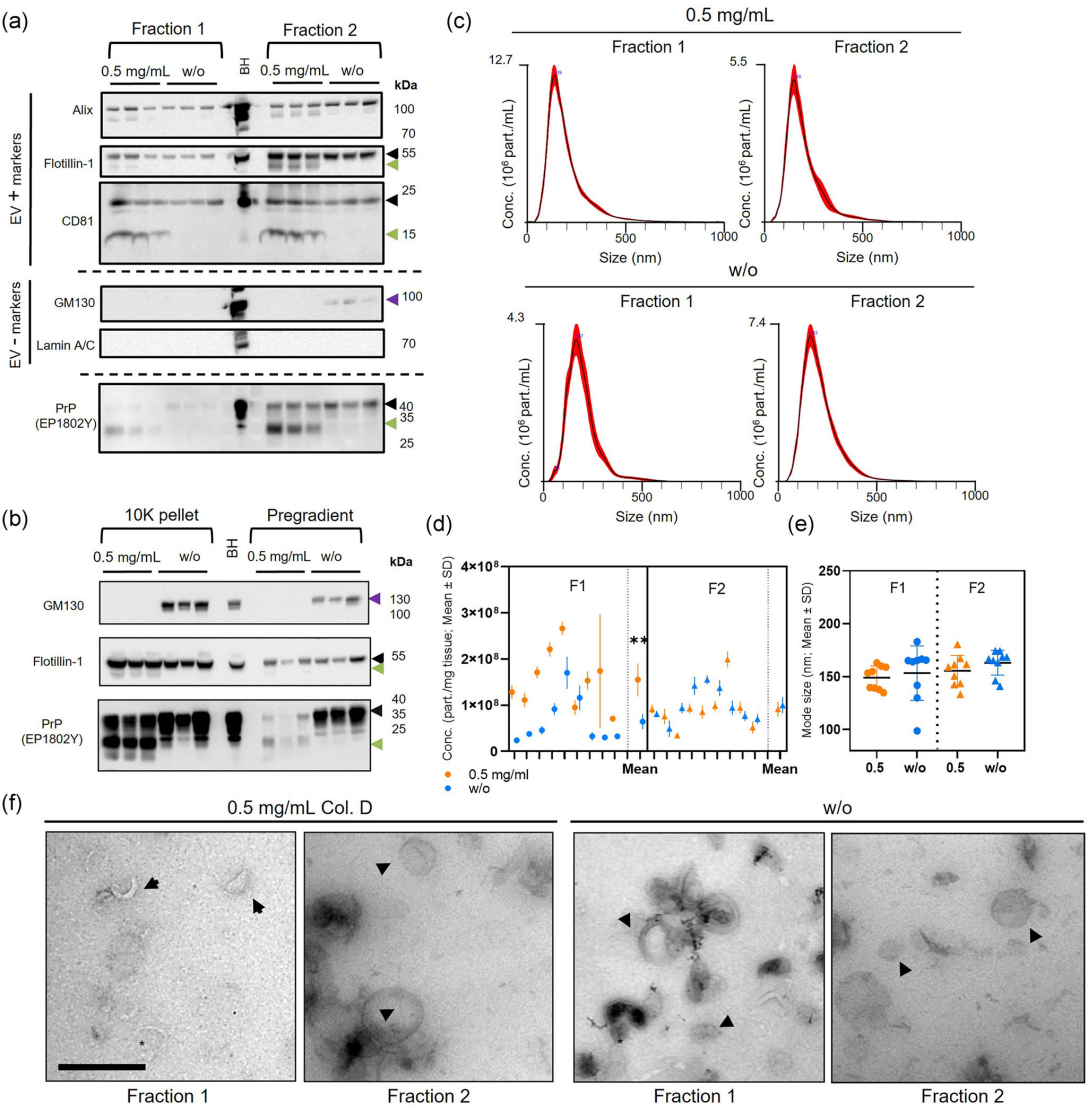
BDEV isolation from mouse brain tissue without enzymatic digestion maintains BDEV purity and prevents artificial proteolytic processes. (a) WB analyses of F1 and F2 BDEVs samples isolated from female mouse brain tissue, with or without the addition of 0.5 mg/mL of collagenase D (n = 3 for each condition), were labelled for PrP^C^, the EV positive markers Alix, Flotillin‐1, and CD81 as well as for the Golgi and nuclear markers, GM130 and Lamin A/C as EV negative markers. The green arrowheads indicate collagenase‐mediated cleavage fragments observed for PrP^C^, CD81, and Flotillin‐1, while the purple arrowhead denotes the unexpected presence of GM130 (in non‐enzyme‐treated F2). A total mouse brain homogenate (BH) served as a control. In (b), the 10,000×g pellet (10K) and the pre‐gradient BDEVs show a total GM130 disappearance when collagenase was used, and PrP^C^ and Flotillin‐1 again displayed the cleavage pattern observed before in BDEVs. (c) Representative size distribution graphs from NTA analysis of F1 and F2 BDEVs show the expected normal‐like distribution. The concentration of particles per mg of initial tissue (d) and mode size analysis in nm (e) of BDEVs obtained with both protocols were measured with NTA (n = 9 for each condition). The particle concentration values are shown as the mean value of each sample measurement with its SD derived from its technical replicates. No differences in the particle mode size were observed between the same fraction in both protocols, but the F1^‐^ displayed significantly fewer particles/mg of tissue than the F1^+^. Data are presented as mean ± S.D. in (d) and (e). (f) TEM images of negative stained BDEVs showing the typical double membrane and cup shape. BDEVs are indicated with arrowheads. Scale bar = 500 nm.

**FIGURE 4:**
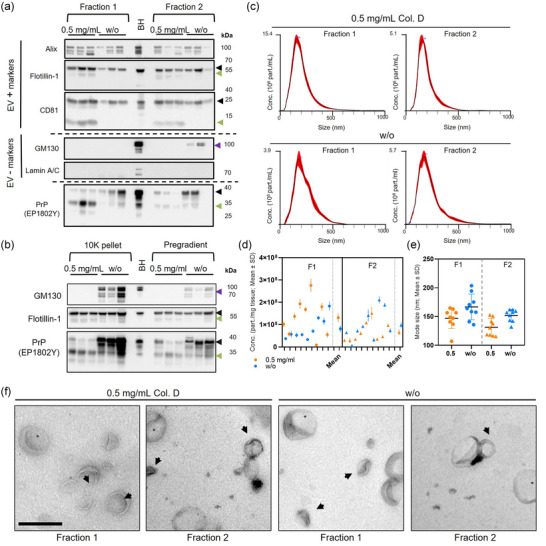
BDEV isolation from human brain tissue without enzymatic digestion maintains BDEV purity and prevents artificial proteolytic processing. (a) Representative WBs of F1 and F2 BDEVs samples isolated from male human brain tissue, with or without the addition of 0.5 mg/mL of collagenase D (*n* = 3 for each condition), for PrP^C^, the EV positive markers Alix, Flotillin‐1, and CD81 and the Golgi and nuclear markers, GM130 and Lamin A/C, as EV negative markers. Green arrowheads indicate collagenase‐mediated cleavages observed for PrP^C^, CD81, and Flotillin‐1, and the “unexpected” bands for GM130 in F2 (w/o collagenase) are indicated by a purple arrowhead. A total human brain homogenate (BH) was used as a loading control. In (b), the 10,000 × *g* pellet (10K) and the pre‐gradient BDEVs show a total GM130 disappearance when collagenase was used, and PrP^C^ and Flotillin‐1 displayed the cleavage pattern observed before in BDEVs. (c) Representative size distribution graphs from NTA analysis of F1 and F2 BDEVs show the expected normal‐like distribution. The concentration of particles per mg of initial tissue (d) and mode size analysis in nm (e) of BDEVs obtained with both protocols were measured with NTA (*n* = 9 for each condition). The particle concentration values are shown as the mean value of each sample measurement with its SD derived from its technical replicates. No differences were observed in the particle concentration between both protocols, however when comparing BDEV from the same fraction isolated with both approaches. Data are presented as mean ± S.D. in (d) and (e). (f) TEM images of negative stained BDEV illustrating the typical double membrane and cup shape. BDEVs are indicated with arrowheads. Scale bar = 500 nm.

To evaluate the efficiency of our protocol in different systems, we isolated EVs from mouse and human brain tissues (male and female in both cases). Each brain tissue sample was cut into two pieces (100–200 mg per piece): one piece was isolated without the addition of collagenase (w/o), while the other underwent isolation with collagenase. The BDEV protein characterization yielded consistent results in both mouse and human samples (Figures 3, 4, and Figures S6 and S7). A clear expression of the positive EV markers (EV+) Alix, CD81 and Flotillin‐1 was observed with both protocols. However, CD81 and Flotillin‐1, as observed in Figure 1, exhibited distinct patterns in the presence of collagenase. The EV‐negative marker Lamin A/C was not detected in any fraction, but GM130 was present in F2 of the BDEVs isolated without collagenase in all cases suggesting that its absence in the collagenase‐treated samples is due to digestion (as observed in Figure 1 for tissue homogenates). Notably, PrP^C^ was predominantly found in F2 of mouse BDEVs but distributed across both fractions of the human BDEVs consistently in both sexes. Regardless of the source, PrP^C^ consistently exhibited cleaved patterns in the enzyme‐treated BDEVs samples, indicating an enriched PrP^C^‐C1‐like fragment (Figures 3, 4, and Figures S6a and S7a) consistent with our observations in collagenase‐digested brain homogenates (Figure 1c and Figure S1). To determine whether the proteolytic changes observed were directly linked to the enzymatic isolation, we checked protein levels in the 10K pellet and the Pre‐gradient BDEVs (Figures 3, 4 and Figures S6b and S7b). The PrP^C^ pattern was similar to the one observed in F1 and F2, with an enriched PrP^C^‐C1‐like fraction in the collagenase‐based BDEVs. GM130 was observed in the enzyme‐free BDEVs samples, but not in the enzyme‐based ones, again suggesting that the absence of GM130 in these samples is due to digestion (Figures 3, 4 and Figures S6b and S7b). As expected, no EV markers were detectable in the F3 and F4 fractions of mouse and human samples (Figures S3 and S4), and only a weak GM130 signal in the mouse BDEV was observed (Figures S3 and S4). Uncropped blots and corresponding total protein staining are presented in Figure S5.

NTA revealed the characteristic size distribution and particle counts between 0.3−6 × 10^8^ particles/mg of tissue in both mouse and human isolates, indicating a comparable BDEV isolation yield with both procedures, independently of the sex or species analysed (Figures 3, 4 and Figures S6c–e, S7c–e and S8). In the case of female mouse BDEVs, there were significantly more particles/mg in the F1 of collagenase samples (F1^+^) compared to the respective enzyme‐free samples (F1^−^), while all other fractions had a very similar number of particles (F1^+ ^= 1.54 × 10^8^ ± 2.06 × 10^7^ part./mg; F2^+ ^= 9.1 × 10^7^ ± 1.52 × 10^7^ part./mg; F1^−^ = 6.4 × 10^7^ ± 1.68 × 10^7^ part./mg; and F2^−^ = 9.9 × 10^7^ ± 1.21 × 10^7^ part./mg; F1^+^ vs. F2^+^: *p* = 0.201; F1^+^ vs. F1^−^: **p = 0.004; F1^−^ vs. F2^−^: *p* = 0.61; F2^+^ vs. F2^−^: *p* > 0.99, Kruskal–Wallis test) (Figure 3d and Figure S8a). However, this significant difference was not observed in male mice BDEVs. Interestingly, we found, with both protocols, a significantly higher amount of particles in F2 compared to F1 in the murine male cases (F1^+ ^= 2.30 × 10^8^ ± 2.26 × 10^7^ part./mg; F2^+ ^= 6.22 × 10^8^ ± 6.16 × 10^7^ part./mg; F1^−^ = 7.25 × 10^7^ ± 1.16 × 10^7^ part./mg; and F2^−^ = 4.69 × 10^8^ ± 4.25 × 10^7^ part./mg; F1^+^ vs. F2^+^: *^#^p* = 0.048; F1^+^ vs. F1^−^: *p* = 0.85; F1^−^ vs. F2^−^: *^##^p* = 0.008; F2^+^ vs. F2^−^: *p* > 0.99, Kruskal–Wallis test) (Figures S6d and S8a).

The NTA analysis also revealed no overt differences when comparing the same fractions in terms of the size of particles, exhibiting the usual size distribution in female samples (F1^+ ^= 149.1 ± 3.7 nm; F2^+ ^= 155.6 ± 4.8 nm; F1^−^ = 153.4 ± 8.5 nm; F2^−^ = 163.1 ± 3.9 nm; F1^+^ vs. F2^+^: *p *> 0.99; F1^+^ vs. F1^−^: *p* = 0.70; F1^−^ vs. F2^−^: *p* > 0.99; F2^+^ vs. F2^−^: *p* = 0.76, Kruskal–Wallis test) (Figures 3e and S8b) and male‐derived samples (F1^+ ^= 143.6 ± 5.0 nm; F2^+ ^= 142.6 ± 3.28 nm; F1^−^ = 151.1 ± 2.05 nm; F2^−^ = 138.11 ± 2.7 nm; F1^+^ vs. F2^+^: *p *> 0.99; F1^+^ vs. F1^−^: *p* = 0.82; F1^−^ vs. F2^−^: *p* = 0.08; F2^+^ vs. F2^−^: *p* > 0.99, Bonferroni's multiple comparison tests, ordinary one‐way ANOVA) (Figures S6e and S8b).

In case of the human brain‐derived samples, on the one hand, no differences were observed in terms of particle concentration of the male BDEVs in any fraction (F1^+ ^= 1.31 × 10^8^ ± 2.78 × 10^7^ part./mg; F2^+ ^= 8.15 × 10^7^ ± 1.68 × 10^7^ part./mg; F1^−^ = 6.55 × 10^7^ ± 1.45 × 10^7^ part./mg; and F2^−^ = 9.77 × 10^7^ ± 2.30 × 10^7^ part./mg; F1^+^ vs. F2^+^: *p* = 0.66; F1^+^ vs. F1^−^: *p* = 0.22; F1^−^ vs. F2^−^: *p* > 0.99; F2^+^ vs. F2^−^: *p* > 0.99, Bonferroni's multiple comparison tests, ordinary one‐way ANOVA) (Figures 4d and S8c). On the other hand, as observed in mice samples, in female human BDEV samples, we obtained significantly more particles in the F1^+^ than in the F1^−^ and F2^+^ (F1^+ ^= 2.22 × 10^8^ ± 1.90 × 10^7^ part./mg; F2^+ ^= 7.30 × 10^7^ ± 2.12 × 10^7^ part./mg; F1^−^ = 1.27 × 10^8^ ± 1.57 × 10^7^ part./mg; and F2^−^ = 5.56 × 10^7^ ± 1.37 × 10^7^ part./mg; F1^+^ vs. F2^+^: ^###^ *p* < 0.001; F1^+^ vs. F1^−^: **p = 0.007; F1^−^ vs. F2^−^: *p* = 0.06; F2^+^ vs. F2^−^: *p* > 0.99, Bonferroni's multiple comparison tests, Ordinary one‐way ANOVA) (Supplementary Figures S7d and S8c). As observed in murine samples, the NTA revealed no size distribution differences in the human BDEVs in both male and female‐derived samples (males: F1^+ ^= 148.6 ± 5.9 nm; F2^+ ^= 131.4 ± 5.0 nm; F1^−^ = 166.8 ± 7.4 nm; F2^−^ = 152.0 ± 3.5 nm; F1^+^ vs. F2^+^: *p *= 0.37; F1^+^ vs. F1^−^: *p* = 0.11; F1^−^ vs. F2^−^: *p* = 0.44; F2^+^ vs. F2^−^: *p* = 0.09, females: F1^+ ^= 139.8 ± 2.72 nm; F2^+ ^= 128.6 ± 7.68 nm; F1^−^ = 145.2 ± 2.94 nm; F2^−^ = 134.5 ± 3.11 nm; F1^+^ vs. F2^+^: *p *= 0.60; F1^+^ vs. F1^−^: *p* > 0.99; F1^−^ vs. F2^+^: *p* = 0.12; F2^+^ vs. F2^−^: *p* > 0.99; both analysed with the Bonferroni's multiple comparison tests, Ordinary one‐way ANOVA) (Figures 4e, S7e and S8d).

Overall, the NTA results demonstrated that the collagenase‐free protocol yields particles with a normal‐like size distribution and concentration of particles per tissue weight similar to the samples isolated with 0.5 mg/mL of collagenase D, except for the female‐derived F1^+^ BDEV samples that showed more particles while isolated with collagenase D, both in mouse and human, suggesting potential minor sex‐related differences that should be explored further (see comparison between sexes and summary in Figure S8).

Lastly, we characterized the isolated BDEVs by labelling them with negative staining (uranyl acetate) and imaged them with TEM (Figures 3, 4, S6f and S7f). We observed the characteristic cup‐shaped and membranous EV structures in both fractions (F1 and F2), with no discernible differences between the protocols or tissue origins. For a comprehensive illustration of the variety of BDEVs obtained across fractions and protocols, please refer to Figure S10, which presents a detailed compilation of TEM images.

In order to further characterize the isolated BDEVs, their protein concentration was analysed using a Micro BCA™ kit. The results showed no differences in protein yield between the BDEVs from the same fraction isolated with both approaches, neither in humans nor mice samples (mouse samples: F1^+ ^= 0.067 ± 0.029 µg/mg; F2^+ ^= 0.331 ± 0.074 µg/mg; F1^−^ = 0.043 ± 0.022 µg/mg; F2^−^ = 0.196 ± 0.061 µg/mg; F1^+^ vs F2^+^: ^#^ *p *= 0.01; F1^+^ vs. F1^−^: *p* > 0.99; F1^−^ vs. F2^−^: ^#^ *p* = 0.048; F2^+^ vs. F2^−^: *p* > 0.99, analysed with Kruskal–Wallis test; human samples: F1^+ ^= 0.055 ± 0.012 µg/mg; F2^+ ^= 0.050 ± 0.014 µg/mg; F1^−^ = 0.049 ± 0.022 µg/mg; F2^−^ = 0.054 ± 0.026 µg/mg—all comparation *p* > 0.99, analysed with the Bonferroni's multiple comparison tests, Ordinary one‐way ANOVA) (Figure S9).

### 3.4 Non‐enzymatic BDEV isolation has minimal impact on the EV‐associated proteome

**FIGURE 5:**
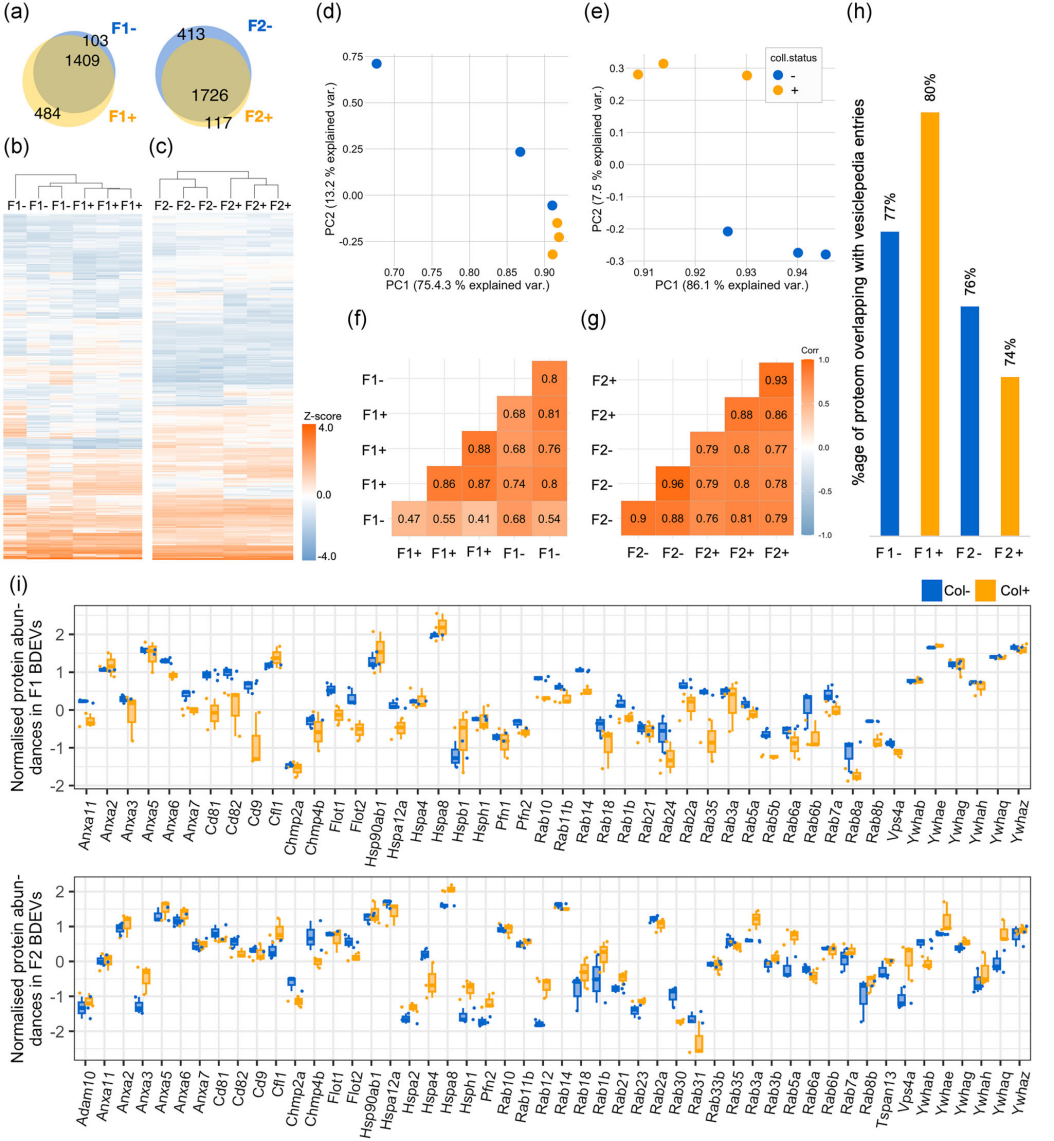
The proteomes of mouse BDEVs isolated using the collagenase‐free method (indicated with ‘−’) and those with the collagenase‐based protocol (labelled as ‘+’) were analysed systematically (*n* = 3, female, for each condition). (a) Venn diagrams display a high overlap in the detected proteins between F1^−^ and F1^+^ as well as between F2^−^ and F2^+^. Heatmaps displaying protein abundances (column *z*‐score) of F1^−^ compared to F1^+^ (b) and F2^−^ versus F2^+^ (c). Scatter plots showing principal component analysis (PCA) of the proteomic composition of mouse BDEVs prepared using the two protocols for F1 (d) and F2 (e). Correlation plots highlight the similarities between F1^−^ and F1^+^ (f) and between F2^−^ and F2^+^ (g); the colour key represents variations in Pearson's correlation coefficient values. Proteins constituting the mouse BDEVs were checked for their overlap with the Vesiclepedia database using the FunRich enrichment software (h). Mouse BDEV samples obtained with collagenase‐free and collagenase‐assisted methods from both F1 and F2 showed high overlaps for their constituting proteins (more than 74%), with the Vesiclepedia database. Graphs showing normalized protein abundances of bona fide EV markers observed in col^+^ and col^‐ ^F1 and F2 BDEVs isolated from female mice (i).

**FIGURE 6:**
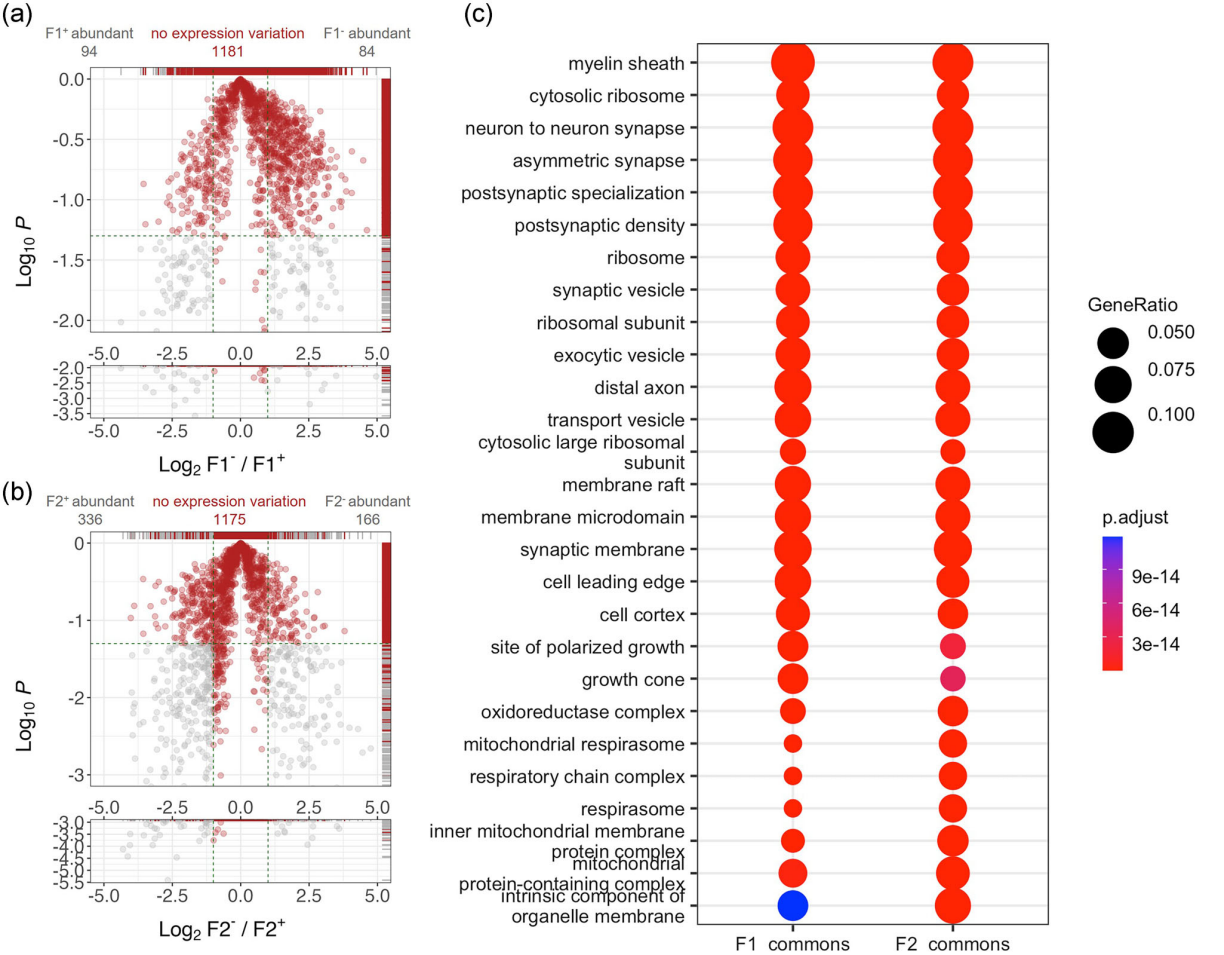
(a) The inverted volcano plot shows the 1,181 proteins commonly expressed with no expression (abundance) differences in F1^−^ compared to F1^+^; 94 proteins were found to be overexpressed, and 84 proteins were found to be downregulated in F1^−^ compared to F1^+^, shown in grey. (b) Inverted volcano plot shows 1,175 proteins commonly expressed with no difference in their relative abundances in F2^−^ and F2^+^ (in red); 166 proteins were found over‐expressed, and 336 proteins were found to be less expressed in F2^−^ when compared to F2^+^. In a and b, the *x*‐axis signifies log2 fold change (F1^−^/ F1^+^). The *y*‐axis shows the log10 of *p*‐values; *y*‐axis values larger than −1.3 (log10 of 0.05) highlight expression difference values with *p*‐values > 0.05 for the pairwise student *t*‐test. The intercepts on the *x*‐axis indicate the cut‐offs for expression changes, set at −1.5‐fold for down‐regulation and +1.5‐fold for up‐regulation. The intercept on the *y*‐axis marks the cut‐off set for −1.3 (log10 of 0.05). (c) Dot plots depicting the top 20 cellular component categories linked to proteins expressed with no significant differences in their abundances between F1^−^ and F1^+^ and between F2^−^ and F2^+^ BDEVs. These categories are based on gene ontology database, and the colour key indicates the adjusted p‐values resulting from the over‐representation analysis performed with EnrichR.

**FIGURE 7:**
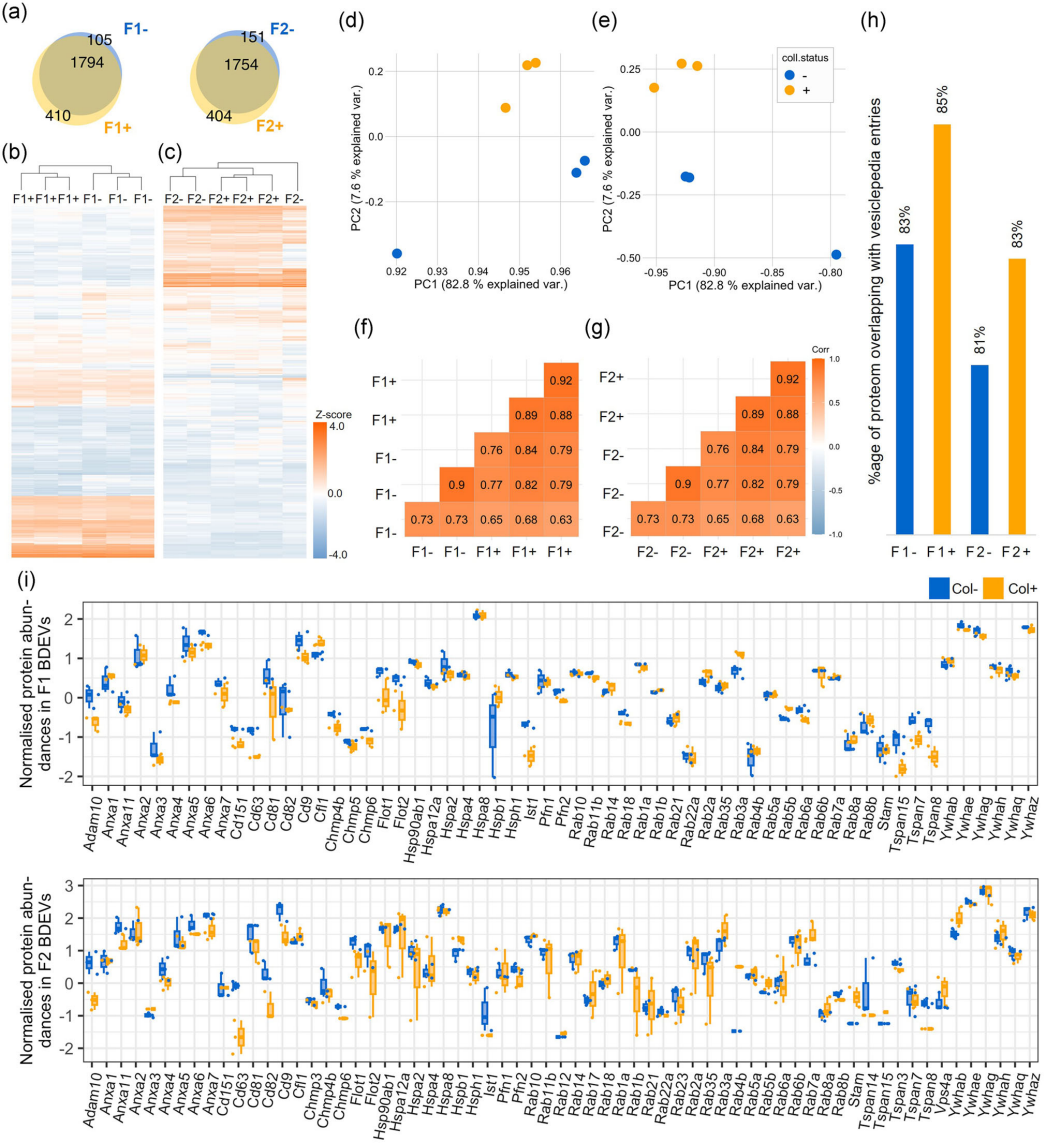
Systematic analysis of human BDEV proteomes: Human BDEVs isolated with or without collagenase show a major compositional overlap. The proteomes of human‐BDEVs isolated using the collagenase‐free method (indicated with ‘−’) and those with the collagenase‐based protocol (labelled as ‘+’) were analysed systematically (*n* = 3, male, for each condition). (a) Venn diagrams display a high compositional overlap between F1^−^ and F1^+^ as well as between F2^−^ and F2^+^. Heatmaps displaying protein abundances (column *z*‐score) of F1^−^ compared to F1^+^ (b) and F2^−^ versus F2^+^ (c). Scatter plots showing principal component analysis (PCA) of the proteomic composition of human‐derived BDEVs prepared using the two protocols for F1 (d) and F2 (e). The correlation plots highlight the similarities between F1 (− vs. +) (f) and F2 (− vs +) samples (g). The colour key represents variations in Pearson's correlation coefficient values. Over 80% of proteins from F1 and F2 EVs from both preparations of human BDEV were found in the Vesiclepedia database (h). Normalized protein abundances of bona fide EV markers observed in col^+^ and col^−^ F1 and F2 BDEVs isolated from female mice (i).

**FIGURE 8:**
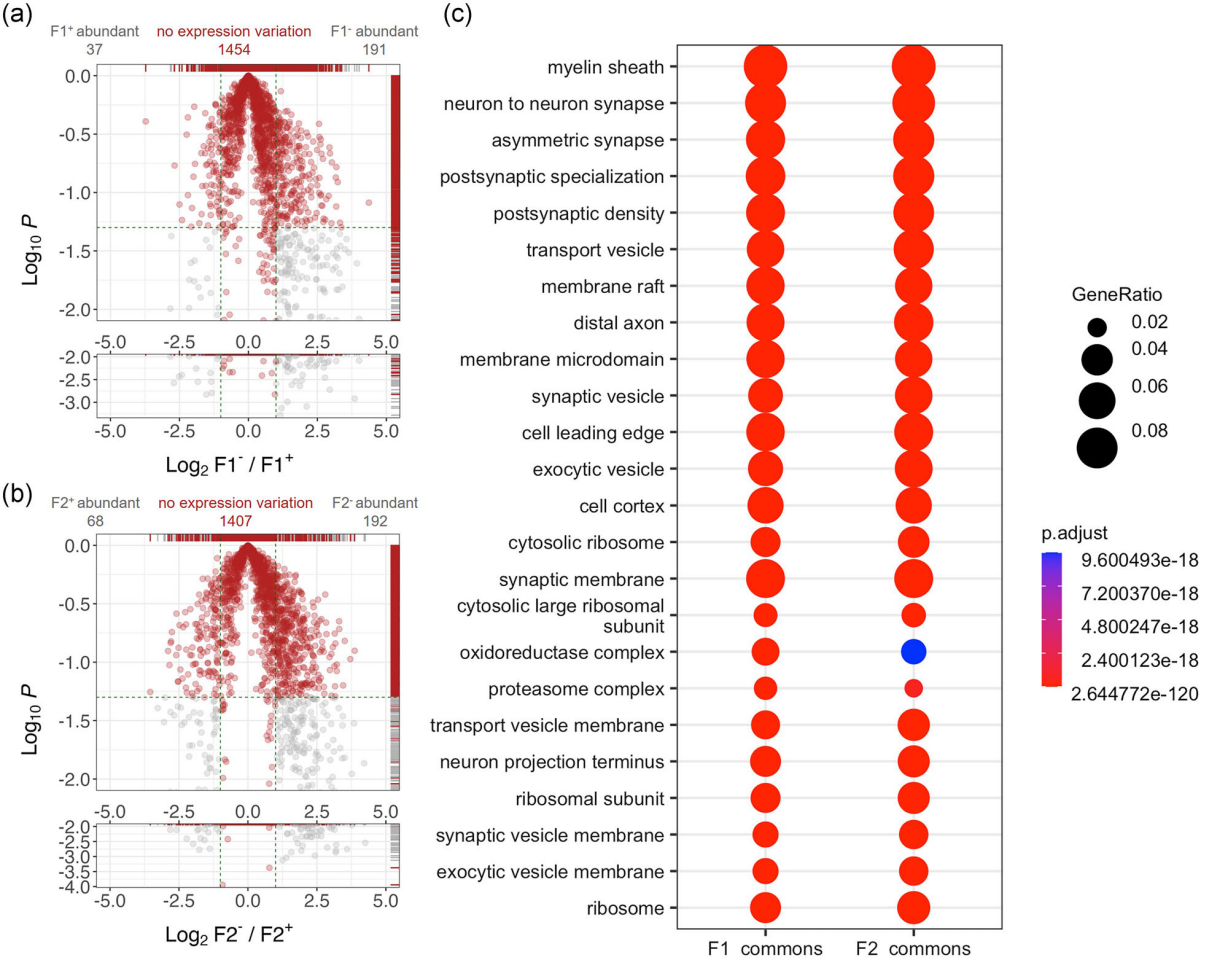
Pairwise comparisons of protein expression patterns highlight a high number of commonly expressed proteins between the col− and col+ human BDEVs, belonging to diverse gene ontology terms. (a) Inverted volcano plot highlighting a big overlap (1,454 proteins with no expression differences, shown in red) between F1^−^ and F1^+^ BDEVs. Relatively under‐represented (37 proteins) and up‐regulated (91 proteins) in F1^−^ are displayed in grey. (b) Inverted volcano plot showing 1,407 proteins commonly expressed with no difference in their relative abundances in F2^−^ and F2^+^; 68 proteins were found to be more abundant, and 192 proteins were found to be less expressed in F2^−^ in comparison to F2^+^. In a and b, the *x*‐axis signifies log2 fold change (F1^−^/ F1^+^). The *y*‐axis shows the log10 of *p*‐values; *y*‐axis values larger than −1.3 (log10 of 0.05) highlight expression difference values with *p*‐values > 0.05 for the pairwise student *t*‐test. The intercepts on the *x*‐axis indicate the cut‐offs for expression changes, set at −1.5‐fold for down‐regulation and +1.5‐fold for up‐regulation. The intercept on the *y*‐axis marks the cut‐off set for −1.3 (log10 of 0.05). (c) Dot plots depicting the top 20 cellular component categories linked to proteins expressed with no significant differences in their abundances between F1^−^ and F1^+^ and between F2^−^ and F2^+^ BDEVs. These categories are based on gene ontology database, and the colour key indicates the adjusted *p*‐values resulting from the over‐representation analysis performed with EnrichR.

To assess whether a similar population of EVs was being isolated with and without collagenase, we conducted a proteomic analysis of BDEVs from both mouse and human brain tissue samples either isolated with 0.5 mg/mL of collagenase D (col^+^) or without enzymatic treatment (col^−^). For the analysis, we paid special attention to proteins known to be located in the EV membrane and in the EV corona. Overall, a high degree of similarity was observed between col^+^ and col^−^ BDEVs from F1 and F2 fractions in both mouse and human BDEVs (Figures 5, 6, 7, 8).

### 3.5 Mouse BDEVs proteome analysis

No major differences were observed for the mouse BDEVs (both F1 and F2) prepared with or without collagenase D, displaying high inter‐group similarities in the proteomic profiles, as depicted by protein abundance heatmaps and associated hierarchical clustering (Figure 5b and c). Out of 1,512 proteins found in F1^−^ and 1,893 found in F1^+^, 1,409 were common in both F1 samples, 103 were just found on the F1^−^, and 484 were unique for F1^+^. In the case of F2, from 2,139 proteins found on F2^−^ and 1,843 in F2^+^, 1,726 were commonly found, 413 were just found in F2^−^, and 117 in F2^+^ (Figure 5a), thus following a similar pattern as F1.

PCA plots of commonly expressed proteins for F1^−^ and F1^+^ (principal component (PC)‐1 and PC‐2 representing 75.4% and 13.2% of data variances, respectively) and correlation plots with high Pearson's coefficient values highlight a high degree of overlap between their proteomic compositions (Figure 5d and f). However, one F1^−^ sample behaved as an outlier, as depicted by lower Pearson coefficient values (Figure 5f). Related to F2^−^ and F2^+^ (with PC‐1 and PC‐2 representing 86.1% and 7.5% of data variances, respectively), the correlation plot also displayed resemblances in the proteomic profiles (Figure 5e and g). To further validate our method and assess the concordance with known EV‐associated proteins, we compared the proteins detected in the F1 and F2 mouse BDEVs samples (both±) with protein entries of the Vesiclepedia database (Chitti *et al*., 2024; Kalra *et al*., 2012; Pathan *et al*., 2019). A high proportion (more than 74%) of the proteins (both F1 and F2) showed an overlap with the Vesiclepedia database protein entries (i.e., F1^−^: 77%; F1^+^: 80%; F2^−^: 76%; and F2^+^: 74%) (Figure 5h).

Utilizing proteome data, we checked for bona fide EV marker proteins (listed in Crescitelli *et al.* (2021) between both F1 and F2 preparations. No substantial expression changes (more than 2‐folds in either direction) were observed in BDEVs isolated with collagenase‐free and collagenase‐based methods, showing that the BDEV populations isolated were similar (Figure 5i).

In pairwise comparisons, we observed a significant overlap between the mouse F1^−^ and F1^+^, that is, 1,181 proteins, with no significant difference in protein abundances (Figure 6a). Likewise, between F2^−^ and F2^+^ mouse BDEVs 1,175 proteins showed no significant differences (Figure 6b). To further compare the physiological profiles of different BDEV populations, we also performed a gene‐ontology (GO) analysis of proteins commonly expressed between the col^−^ and col^+^ EVs (Figure 6c and Supplementary data 1). Commonly expressed proteins in both F1 (col^+^ and col^−^) as well as F2 (col^+^ and col^−^) BDEVs were associated with various cellular components. The top‐20 GO categories majorly included myelin sheath (GO:0043209), synapses related proteins (GO:0098984, GO:0032279, GO:0099572, GO:0014069, GO:0008021 and GO:0097060), membrane microdomains / rafts (GO:0097060 and GO:0045121), growth cones (GO:0030426 and GO:0030427), ribosomes (GO:0005840 and GO:0044391), different type of vesicles (GO:0008021, GO:0070382 and GO:0030133) and mitochondrial components (GO:1990204, GO:0005747, GO:0030964, GO:0045271, GO:1902495 and GO:1990351).

Among the proteins differentially associated to either the col^+^ or col^−^ F1 and F2 EVs, 84 proteins were found to be relatively enriched in F1^−^, while 94 proteins displayed lower expression in the F1^−^ samples (Figure S11a). Most obvious differences in the GO enrichment terms associated with the F1^−^ abundant and F1^+^ deficient proteins are highlighted in a comparative analysis (Figure S11c). F1^−^ BDEVs from mice contained a group of proteins related to nuclear paraspeckles (GO:0016607 and GO:0042382), and various mitochondria‐related GO terms (GO:0098800, GO:1990204, GO:0005746 and GO:0098803). However, the most differentiating groups of GO terms associated with F1^+^ BDEVs, included ER and organelle membrane‐related proteins (GO:0140534, GO:0031301 and GO:0031300), and synaptic components (GO:0097060).

In the context of F2^−^ versus F2^+^ comparison, 166 proteins were identified to be significantly more abundant in F2^−^, and 336 proteins were found to be significantly under‐represented in F2^−^ compared to F2^+^ BDEVs S11b). Comparative GO analysis showed that the upregulated proteins in the F2^−^ were distinctly associated to the following GO terms: distal axon (GO:0150034), site of polarized growth (GO:0030427), and synaptic components (GO:0097060, GO:0098984), growth cone (GO:0030426); and GO terms exclusive to F2^+^ BDEVs included collagen‐containing extracellular matrix (GO:0062023), Golgi apparatus sub‐compartment (GO:0098791), inclusion body (GO:0016234), and cytoplasmic ribonucleoprotein granule (GO:0036464) (See Figure S11d and Supplementary data 1).

### 3.6 Human BDEVs proteome analysis

In the case of human BDEVs, hierarchical clustering of the proteomic profiles of the BDEV isolated with the two methods and in both F1 and F2, described a close inter‐group similarity, as shown in the protein abundance heatmaps (Figure 7b and c). Out of 1,899 proteins identified in F1^−^ and 2,204 found in F1^+^, 1,794 were common in both F1 samples, 105 were exclusively found in F1^−^, and 410 were unique for F1^+.^ In the case of F2, similar trends were found, from 1,905 detected proteins in F2^−^ and 2,158 in F2^+^, 1,754 were commonly found, 151 were just in F2^−^, and 404 in F2^+^ (Figure 7a). In the PCA, BDEVs from F1 (F1^−^ and F1^+^) and F2 (F2^−^ and F2^+^) exhibited close clustering (Figure 7d and e) along with high Pearson's correlation coefficient values (Figure 7f and g), highlighting that only subtle differences could be observed among the collagenase‐free and collagenase‐based preparations.

As for the mice BDEVs, we also checked the concordance of human BDEVs proteomes with the protein database entries of Vesiclepedia. Here, we found that over 80% of the proteins detected in F1^−^ (83%) and F1^+^ (85%) as well as F2^−^ (81%) and F2^+^ (83%), have been previously reported to be associated with EVs (Figure 7h). As observed with the mouse‐derived BDEVs, no substantial expression variations for the bona fide EV marker proteins were found between the col^−^ and col+ human BDEVs in both the F1 and the F2, validating again the isolation of similar BDEV populations (Figure 7i).

We observed 1,454 proteins between the F1^−^ and F1^+^ BDEVs (Figure 8a) and 1,407 proteins between F2^−^ and F2^+^ BDEVs did not show any expression differences (Figure 8b). The GO over‐representation analysis of these proteins highlighted a high degree of association to various synaptic components (GO:0098984, GO:0032279, GO:0099572, GO:0014069, GO:0042734 and GO:0097060), different types of vesicles, such as exocytic vesicles, transport vesicles, and synaptic vesicles (GO:0030133, GO:0008021 and GO:0070382), oxidoreductase complexes (GO:1990204) and myelin sheath (GO:0043209) in both F1^−^ and F1^+^ as well as F2^−^ and F2^+^ BDEVs (Figure 8c and Supplementary data 2).

Among the proteins differentially associated to the col^+^ and col^−^ F1 and F2 BDEVs, we found that there were 191 proteins relatively more abundant in the F1^−^, and 37 were relatively downregulated in F1^−^ compared with the F1^+^ samples (Supplementary Figure S12a). In the comparative GO enrichment analysis, we observed that the top GO terms from F1^+^ and F1^−^ BDEVs were actually quite distinctly associated with the respective groups. GO terms including postsynaptic specialization (GO:0099572), postsynaptic density (GO:0014069), transport vesicles (GO:0030133), asymmetric synapse (GO:0032279), and synaptic vesicle (GO:0008021) were predominantly associated to the human F1^−^ BDEVs and GO terms linked to mitochondrial components (GO:0098800, GO:0098798, GO:0005743, GO:0098803, GO:0005746, GO:0070469) were distinctively associated to F1^+^ BDEVs (Supplementary Figure S12c). Likewise, 192 proteins were significantly upregulated in F2^−^, and 68 proteins were downregulated in F2^−^ compared to F2^+^ BDEVs (Supplementary Figure S12b). In the case of F2 BDEVs, the comparative enrichment analysis highlights distinct functional categories associated with the F2, included postsynaptic specialization (GO:0099572), postsynaptic density (GO:0014069), asymmetric synapse (GO:0032279), synaptic vesicle (GO:0008021), and neuron to neuron synapse (GO:0098984). GO terms distinctly associated to the F2^+^ BDEVs were distinctly related to mitochondria and respiratory chain complexes (GO:0098800, GO:0005743, GO:0098798, GO:0098803 and GO:0005746). BDEVs proteins associated with these different categories are shown in Figure S12d (see also Supplementary data 2).

### 3.7 Transcript screening of mice and human BDEVs reveals no differences in mRNA cargo among the BDEVs enriched with enzyme‐free and collagenase‐based methods

**FIGURE 9:**
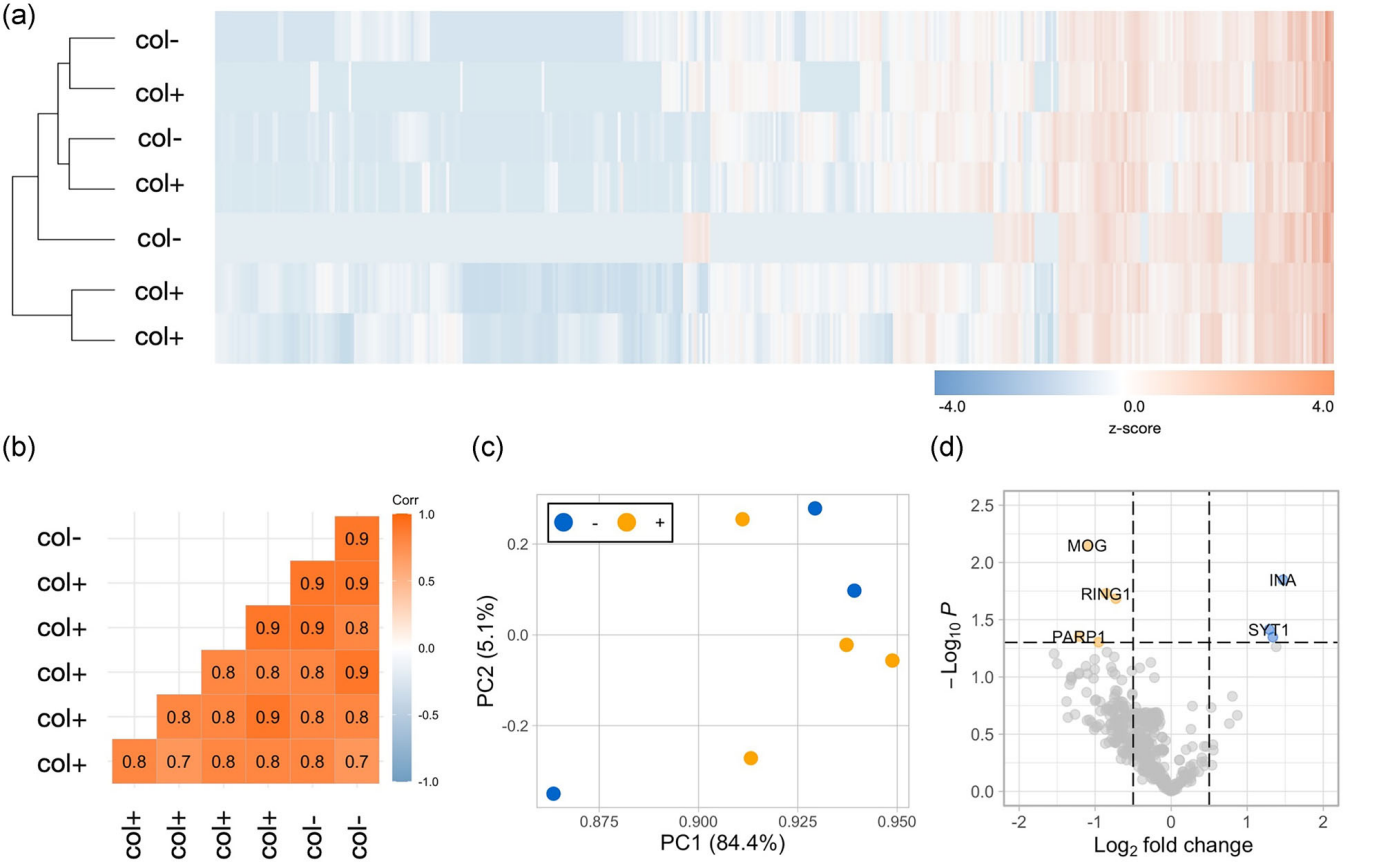
(a) Heat map displaying mRNA expressions (*z*‐score) of col^−^ (male samples, *n* = 3) and of col^+^ samples (*n* = 4) assessed by use of the Nanostring nCounter® Neuropathology panel without previous mRNA purification. (b) Correlation plot highlighting the similarities between samples isolated with collagenase‐free and collagenase‐based protocols. The colour key represents the variation in Pearson's correlation coefficient values. (c) Scatter plots showing principal component analysis (PCA) of mRNA expression profiles of col^−^ and col^+^. (d) Volcano plot highlighting the downregulated (shown in orange) and upregulated (shown in blue) mRNAs in the BDEV preparations. The *x*‐axis intercepts denote the thresholds for expression changes, established at 1.5‐fold for down‐regulation and 1.5‐fold for up‐regulation. The *y*‐axis intercept signifies the threshold set for a *p*‐value of 0.05.

With the previous experiments, we demonstrated that the isolated BDEV populations with and without collagenase were similar in terms of protein composition. To further investigate similarities/differences among the BDEV preparations, we then employed the Nanostring nCounter^®^ Neurodegeneration panel to assess mRNA composition using the same protocol as previously described (i.e., omitting a previous mRNA purification step; Bub *et al*., 2022). For the analysis, we pooled F1 and F2 of human BDEVs isolated either with (0.5 mg/mL) or without collagenase. Heat maps and correlation plot analysis show no significant differences in mRNA expression profiles between collagenase‐free and collagenase‐based BDEVs since they did not cluster according to isolation procedure (Figure 9a and b). The PCA plot likewise indicates an overall similarity in the mRNA expression profiles of the data, although one of the collagenase‐free samples was considered an outlier (Figure 9c). In essence, only two mRNA transcripts, *INA* and *SYT1*, were overrepresented, while three transcripts, *MOG*, *RING1*, and *PARP1*, were found to be deficient in the BDEVs purified without collagenase (col^−^), as depicted by the volcano plot (Figure 9d). In sum, these similar mRNA profiles add to the lack of major differences presented above with regard to EV size, abundance, and protein composition while avoiding artificial protein cleavage and, thus, further validate the suitability of the enzyme‐free isolation protocol presented here.

## 4 DISCUSSION

In this study, we further investigated our observation that collagenase‐based BDEV isolation may lead to artificial cleavage of PrP^C^ (Brenna *et al*., 2020), to other EV markers such as CD81 and Flotillin‐1. Our experiments involving collagenase treatment on mouse and human brain homogenates, on BDEVs, and on N2a‐EVs confirm that the altered protein cleavage can be directly attributed to collagenase treatment. Our approach was to use a recently published protocol to isolate BDEVs from tissue with some modifications (Crescitelli *et al.*, 2021) and compare samples isolated with a lower amount of enzymatic digestion with collagenase D (0.5 mg/mL) or with no applied enzymatic activity, aiming for a protocol that would yield tissue derived EVs with a protein composition as native as possible. We could demonstrate that, even without using collagenase D, we could still isolate BDEVs with high yield and purity, with the advantage of preserving protein integrity on the EV membrane. Our proteomic and mRNA expression analysis further validated that this approach yields the BDEV population with a high compositional overlap with those obtained with low collagenase‐based isolation. However, it remains to be investigated whether BDEVs populations isolated with enzyme‐free method are also similar to those obtained using other isolation methods, that is, employing other proteases and protease concentrations.

One of the initial interactions that EVs engage in after biogenesis is with the ECM, where EVs gain additional surface proteins (the so‐called corona) through various chemical and biophysical interactions, including hydrogen and covalent bonding (Debnath *et al.*, 2023). This corona bestows the EVs with different biological properties (Tóth *et al.*, 2021; Wolf *et al.*, 2022). One of the key considerations in EV isolation from tissue is the trade‐off between efficiently releasing EVs from the ECM while avoiding intense cell and EV breakage, which can contaminate the EV preparations with cellular debris as well as intracellular vesicles, and vesicle‐like structures such as endosomes, lysosomes, Golgi‐derived vesicles, and nuclear fractions. Also, intracellular/cytosolic components might be released and could then unspecifically stick to the EV corona. For this reason, it is crucial to avoid a harsh tissue homogenization process and include a specific separation procedure to separate the EVs from the cell debris (e.g., density‐based gradients and differential centrifugation). EV isolation from brain tissue typically includes the use of proteases of different types to help in this process, such as papain (D'Acunzo *et al.*, 2022, 2021, 2024, 2023; Gomes *et al.*, 2023; Hurwitz *et al.*, 2018; Pérez‐González *et al.*, 2017), and collagenases type III (Brenna *et al.*, 2020; Bub *et al.*, 2022; Huang *et al.*, 2020; Huang, Arab *et al.*, 2023; Su *et al.*, 2021; Vella *et al.*, 2017; Zhang *et al*., 2023), to break down the associations of EVs with the ECM and release them (Debnath *et al.*, 2023; Vella *et al.*, 2017). Here, we tested collagenase type D, which has a minimal effect on cells and has been described as efficient in isolating EVs from cancer‐derived tissue (Crescitelli *et al.*, 2020, 2021). However, these proteases, due to their off‐target proteolytic activity, could also induce unwanted protein cleavages (Duarte *et al*., 2016), leading to degradation or trimming of EV‐associated proteins and possibly affecting both their downstream experimental analysis and physiological behaviour. Others have recently addressed the need to use non‐enzymatically based approaches to obtain BDEVs. On the one hand, Gomes *et al*. studied the possibility of isolating EVs spontaneously released by dissected brain tissue incubated in a culture medium, and they compared them with BDEVs isolated by tissue dissection and digestion with papain, differential centrifugation, and a density gradient. Interestingly, EVs obtained with the ‘release method’ displayed a purer protein profile and a clear EV structure by cryo‐EM. However, the protein yield was reduced, and also their mean size was smaller compared to the tissue‐purified ones (Gomes *et al.*, 2023). On the other hand, Pait *et al.* isolated EVs from hippocampal interstitial fluid using in vivo microdialysis. Pait *et al.* also compared these EVs with the ones isolated by size exclusion chromatography (SEC), ultracentrifugation, or precipitation, and merely observed EV size differences. Unfortunately, WB analysis was not done in that study, so we cannot compare their study directly with our data (Pait *et al*., 2024). These works, and also the one recently published by Zhang *et al*. where BDEVs isolated using density‐gradient ultracentrifugation, SEC, and affinity capture (even though all after collagenase type III digestion) are compared (Zhang *et al*., 2023), together with our work here demonstrate the need in the field to improve and validate further BDEVs isolation methods that reduce their impact on the downstream experiments.

Enzymatic dissociation is the method of choice for preparing primary cell cultures from tissues and has been studied in detail to ensure the perfect balance between the optimal isolation of cells and minimal artificial modifications (Autengruber *et al*., 2012). For instance, previous studies have explored the effects of such enzymatic‐based isolation protocols in various cell types and tissues: satellite stem cells from muscle tissue (Miersch *et al*., 2018), mesenchymal stem/stromal cell isolation from umbilical cord tissue (Taghizadeh *et al*., 2018), adipose‐derived stem cells (Koellensperger *et al.*, 2022), chondrocytes from cartilage (Hidvegi *et al.*, 2006), T cells from spleen and lung tissue (Liu *et al*., 2018) and neurons and glial cells from brain cortex (Hu *et al.*, 2022; Mattei *et al*., 2020; Panchision *et al.*, 2007). Similarly, O'Flanagan *et al.* reported that the collagenase digestion for dissociating solid tumour tissues triggers a stress response that alters the transcriptome of the isolated cancer cells (O'Flanagan *et al*., 2019). In line with this, Mattei *et al.* described that the enzymatic digestion of fresh brain tissue induces critical and consistent alterations in the proteome and transcriptome of neuronal and glial cells (Mattei *et al.*, 2020). These studies highlight the impact of diverse enzyme‐based cell isolation protocols on surface proteins that critically affect downstream flow cytometry analysis (Liu *et al.*, 2018; Mattei *et al.*, 2020; O'Flanagan *et al*., 2019; Taghizadeh *et al.*, 2018). This relates to our findings concerning EV‐membrane proteins such as CD81 and PrP^C^ and demonstrates the relevance of enzyme‐free isolation.

We observed similar results in all the models (human/mouse and female/male) regarding the overall protein content analysed by WB regardless of the protocol used. In the mouse F2 samples, we observed a higher expression of the EV markers Alix, Flotillin‐1, and PrP^C^ than in the F1 in both sexes. In the same way, in the human BDEVs, the EV markers CD81, Flotillin‐1, and Alix were equal in both fractions, but PrP^C^ was seemingly more associated with F1 BDEVs. Moreover, we also observed the same artificial protein cleavages in CD81, Flotillin‐1, GM130, and PrP in all cases when BDEVs were isolated with collagenase, validating its role in artificial protein pruning.

In case of CD81, we found a second band in the WB analysis of col^+^ BDEVs, which is not observable when BDEVs are isolated using the enzyme‐free protocol. A reduction of CD81 signal in flow cytometry analysis of EVs treated with collagenase D was described in Crescitelli *et al.*, thus reinforcing our results (Crescitelli *et al.*, 2021). The concrete role of CD81 on EVs is still not fully understood, and it seems to be dependent on the cellular origin of the vesicles. However, CD81 has been related to crucial intracellular processes such as immune activation (e.g., CD81 forms a complex with the receptor CD9 that is critical for B cell development and activation) (Fan *et al.*, 2023; Susa *et al*., 2021; Toribio & Yáñez‐Mó, 2022), functions that could be affected by its pruning when isolating the EVs with enzymes. Similarly, the enzymatic digestion could also explain the double band observed for Flotillin‐1 in col^+^ BDEVs, although we did not observe this effect in the digested brain homogenate. This may suggest a lower affinity of the collagenase D to digest this protein in a highly concentrated sample like brain homogenates (crowded with other protein substrates) compared to the purified EV fraction, where Flotillin‐1 is highly enriched. Unfortunately, so far, in only one reported study where BDEVs were isolated with an enzymatic‐free method, CD81, and Flotillin‐1 expression patterns were likewise assessed by WB. In that case, Gomes *et al.* showed a clear non‐digested CD81 and Flotilin‐1 protein expression profile of the spontaneously released EVs, but no noticeable CD81 signal was detected in the papain‐treated BDEV samples, which may be pointing towards out‐target papain proteolysis (Gomes *et al.*, 2023).

Our research focus is on the role of PrP^C^ on EVs in the context of neurodegenerative diseases (Falker *et al.*, 2016; Heisler *et al*., 2018). Therefore, it was crucial for us to study how to preserve PrP^C^’s integrity in BDEVs. PrP^C^ is a GPI‐anchored membrane protein, it is also found in the extracellular space, on EVs’ membrane, and as soluble forms (Matamoros‐Angles *et al*., 2023). The first evidence of PrP^C^’s presence on EVs was described almost two decades ago (Fevrier *et al*., 2004). After that, several publications have shown the important role of EV‐PrP^C^ in many functions related to brain pathophysiology, such as neuroprotection after ischemic conditions (Guitart *et al*., 2016), lysosomal‐exosomal trafficking in neurons (Heisler *et al*., 2018), cellular EV uptake (Brenna *et al*., 2020; D'Arrigo *et al*., 2021), and neurite outgrowth (Gonias *et al*., 2022). Other publications have further validated the relevance of PrP^C^ in EV biology in the context of AD and cancer. Johansson *et al.* showed that the absence of PrP^C^ produced an upregulation of the ESCRT‐dependent proteins VPS25 and Tsg101, an increase in total EV secretion, and an accumulation of Aβ in the cell rather than in the EVs (Johansson *et al*., 2024). Another publication suggested that EVs containing PrP^C^ modulate the progression and tumorigenicity in colorectal cancer (Yun *et al*., 2021).

PrP^C^ experiences endogenous post‐translational modifications and cleavages that generate several physiological PrP forms and fragments, both membrane‐anchored and released to the extracellular space, that likely exert different functions. The two most frequent processes are the α‐cleavage in the central part of the protein, produced by a still unknown protease, generating two structurally different parts, and the shedding of PrP^C^, which is mediated by ADAM10 (see (Linsenmeier *et al*., 2017; Mohammadi *et al*., 2023)). The enzymatic digestion induced by collagenase in brain homogenates appears to be strikingly similar to the naturally occurring α‐cleavage (in the sense that a N‐terminal fragment of ∼10 kDa and a C‐terminal one of ∼15 kDa are formed), yet an additional larger fragment is also formed in the case of collagenase treatment. At present, we cannot judge whether this observed additional fragment results from an alternative cleavage or represents an intermediate form generated in a two‐step process. However, collagenase induces an α‐cleavage‐like processing of PrP^C^ on EVs (thereby further increasing the amount of a PrP^C^‐C1‐like fragment on EVs, which is already physiologically enriched in some EV populations (Brenna *et al*., 2020). Moreover, the artificial modification by collagenase observed here would induce the release of the N‐terminal part of PrP^C^, which typically interacts with diverse ligands, potentially modulating their functions (Shafiq *et al*., 2022), such as synaptic receptors in epilepsy (Carulla *et al*., 2011), toxic Amyloid‐β oligomers in AD (Falker *et al.*, 2016; Halipi *et al.*, 2024; Laurén *et al.*, 2009; Resenberger *et al.*, 2011), α‐synuclein in PD models (Urrea *et al.*, 2018) or the pathogenic misfolded isoform of PrP (PrP^Sc^) in PrD (Turnbaugh *et al*., 2011). Moreover, lack of this N‐terminal part could also affect the interaction of EVs with recipient cells and their uptake since PrP^C^ seems to be involved in modulating EV uptake (Brenna *et al*., 2020). Therefore, any functional analysis aiming to study PrP^C^ on EVs needs to ensure the physiological composition of the various PrP forms, avoiding any undesired proteolytic processes to preserve their function completely and prevent any bias or confusion in downstream experiments.

Surprisingly, we observed a GM130‐positive signal when BDEVs were isolated without enzymes in mouse and human BDEVs. Our results with brain homogenates treated with collagenases (III and D) demonstrate that the GM130 ‘disappearance’ is directly connected to collagenase digestion. Moreover, in our isolation controls, the 10K pellet (containing large EVs) and the pre‐gradient BDEVs, already lack GM130 expression, highlighting that the digestion occurs prior to the downstream BDEV purification steps. However, GM130 was not detectable in EVs isolated from culture supernatants of N2a cells. One possible explanation is that GM130 is released during tissue preparation from disrupted cells, sticking to the EV corona as a contaminant, and that collagenase then degrades extracellularly bound GM130. The fact that we do not detect GM130 in EVs isolated from conditioned media of different cultured cell lines (without significant cell disruption and not using collagenase) or in the isolation of EVs from blood (data not shown), would speak more in this direction. However, given the fact that the presence of GM130 in/on EVs has been described in at least 20 entries of mass spectrometry data in Vesiclepedia (Kalra *et al*., 2012; Pathan *et al*., 2019), we cannot completely rule out the possibility that, in some instances, GM130 can be present in some EV subpopulations. Unfortunately, no GM130 expression analysis by WB was performed in previous studies using enzyme‐free BDEV isolation that would enable us to compare our findings (Gomes *et al.*, 2023; Pait *et al.*, 2024).

In terms of particle size analysed by NTA, we observed the usual normal‐like size distribution in all cases, with no overt differences between the same fraction when comparing the BDEVs isolated with both approaches. When analysing BDEV concentration, the overall particle count in both F1 as well as F2, in mice and human BDEV, was comparable using both protocols. In male mouse BDEVs, both isolation protocols showed a significantly higher particle count in F2 compared to F1, consistent with the observed protein concentrations. However, NTA of female‐derived samples (both mouse and human) revealed a higher concentration of BDEVs in F1 isolated with collagenase compared to F1 isolated without collagenase, suggesting potential sex‐specific differences in overall EV composition in the brain. Additionally, in human female samples, the particle count was higher in F1 than in F2, with significant differences observed only in collagenase‐isolated BDEVs. This suggests that the collagenase‐based method may isolate more low‐density particles, which are more likely to end up in the less dense F1 fraction.

Interestingly, sex‐based differences in BDEVs (concentration, size, or cargo) have already been described by several other groups in different models (Noren Hooten *et al*., 2022). Observed differences in the concentrations of BDEVs from female mice isolated at different ages but not in the male (Kim *et al.*, 2022). Also described larger BDEV sizes, along with impaired EV biogenesis in female rats after nicotine administration (Koul *et al.*, 2020). Obtained more EVs from the hippocampal interstitial fluid of adult male mice than in younger ones, but not from the female mice (Pait *et al.*, 2024. This variability could possibly be explained by the sex‐based differences in brain lipids, which may impact the BDEVs’ lipidic cargo as well (Acaz‐Fonseca *et al.*, 2017; Koul *et al.*, 2020). However, inferences should be taken carefully from the NTA‐based measurements (considering the large intrinsic error margins associated with the particle‐tracking‐based methods) and experimental data should further be reevaluated using other, more precise techniques (Bachurski *et al.*, 2019; Vestad *et al.*, 2017).

Global proteomic analysis highlights a significant overlap between BDEVs of the same density fraction, isolated with and without collagenase D. For corresponding pairs, such as F1^−^ and F1^+^, F2^−^ and F2^+^ BDEVs from both mice (female) and human (male) tissues, the overlap in proteomic composition was over 80%. This indicates that enzyme‐free and enzyme‐assisted protocols do not result in completely different populations of BDEVs. For further validate our method, we assessed the concordance of the BDEV proteomes with known EV‐associated proteins entries of the Vesiclepedia database (Chitti *et al.*, 2024; Kalra *et al*., 2012; Pathan *et al.*, 2019). A high proportion (more than 74%) of the proteins from mice and human BDEVs overlap with the entries in the Vesiclepedia database, regardless of the method used. This highlights the comparable efficiency of the BDEVs enrichment without the collagenase D. With the same goal, we also compared the protein abundances of some bona fide EV markers (Crescitelli *et al.*, 2021) from our proteomics data and, expectedly, observed the markers to be similarly expressed in the BDEVs from mice and human, regardless of the isolation protocol used, further highlighting the similarity of the col^+^ and col^−^ BDEVs.

On the global proteome scale, taking into account commonly expressed proteins in the col^−^ and col^+^ EVs, we also observed no major differences in the composition of BDEVs from F1 and F2 fractions from mice as well as human tissues. In mice, F1 BDEVs 87% proteins, and in F2 BDEVs 70% of proteins showed no significant expression differences. Similarly, human F1^−^ and F1^+^ BDEVs shared 86% of their proteome, while F2^−^ and F2^+^ BDEVs shared 84%, with no expression differences observed.

Upon GO analysis, we found that commonly expressed proteins from mouse BDEVs were primarily associated with several nervous tissue‐related components, such as the myelin sheath, mitochondrial constituents, synapses, membrane microdomains, ribosomes, and various types of vesicles. In the case of human BDEVs, the GO analysis revealed similar categories, including the myelin sheath, various synaptic components, mitochondrial oxidoreductase complexes, transport and exocytic vesicles, and ribosomes. The similar GO profiles, particularly for mitochondrial components, synapses, and myelin (i.e., neuronal and oligodendrocyte‐derived proteins), may indicate the presence of common EV subpopulations isolated with both protocols.

One of the major aspects highlighted by the GO analysis is the presence of mitochondrial components, specifically in mouse BDEVs but also in BDEVs derived from humans. The release of mitochondrial components by cells into the extracellular environment has been widely observed in cultures of endothelial cells (Coly & Boulanger, 2019; Puhm *et al.*, 2019). More recently, a new class of metabolically active EVs, termed mitovesicles, containing majorly mitochondrial components has been identified (D'Acunzo *et al.*,^2021^, ^2022^). These mitovesicles were also found altered in BDEVs from the brain of Down syndrome patients and respective mouse models (D'Acunzo *et al.*, 2024, 2021) as well as after chronic cocaine exposure (D'Acunzo *et al.*, 2023). The presence of high amounts of mitochondrial proteins in both mouse and human BDEVs (col^‐^ and col^+^) may be an indicative of a high proportion of mitovesicles as a constituent of the EV populations in our study, as well. Another important group of proteins highlighted in mouse and human BDEVs equally by GO analyses is synapse‐related proteins. Various classical synaptic markers are reported to constitute the EVs according to the Vesiclepedia database (Chitti *et al.*, 2024). EVs of neuronal origin from primary cells and enriched from plasma contain synaptic proteins (Antoniou *et al.*, 2023; Delgado‐Peraza *et al.*, 2023; Solana‐Balaguer *et al.*, 2023); likewise, BDEVs from human and mouse brain also contain many synaptic proteins (Ladakis *et al.*, 2024; Muraoka *et al*., 2020; You *et al.*, 2022). Finally, the GO terms related to myelin sheath are highlighted by the over‐representation analysis in the BDEV from both species, which mainly constitutes further proteins from oligodendrocyte, cytoskeleton, axons, and etc. Nearly all the proteins constituting the GO term (96% in both mouse and human BDEVs) are reported in the proteins database of Vesiclepedia (Chitti *et al.*, 2024). In summary, it can be inferred that the substantial appearance of mitochondrial, synaptic, and myelinic components in our proteomic data is due to the presence of the mitovesicles and neuronal/oligodendrocytic EVs in our preparations.

On the other hand, we also observed minor differences in the proteomes of col^−^ and col^+^ BDEVs from mice and human as well. In mouse BDEVs, proteins abundant in F1^−^ were related to mitochondria and nuclear paraspeckles, whereas those in F1^+^ were prominently associated with the endoplasmic reticulum and synapses. Proteins abundant in F2^−^ BDEVs exhibited a strong synaptic profile compared to F2^+^, which was related to ECM proteins and Golgi complex proteins. In case of human BDEVs, differentially associated proteins from both F1 and F2 showed similar trends. F1^−^ and F2^−^ BDEVs predominantly contained synapse‐related proteins, while F1^+^ and F2^+^ BDEVs predominantly contained mitochondrial proteins. Taken together, the minor observed proteomic differences in BDEVs composition can conceivably be attributed to the isolation of partially different EV populations in collagenase D‐assisted protocols, owing to higher tissue dissociation and better detachment of EVs from the ECM. Moreover, the proteome variations may occur due to modifications (possibly proteolysis) of proteins of the EV surface and corona when using collagenase, which may affect the peptide populations detected during proteomic analysis.

Besides yielding largely unaltered proteomics results, our enzyme‐free and enzyme‐assisted protocols obtained EV populations with no major differences in mRNA content. EVs have been discussed as major carriers of all kinds of different RNA molecules, especially intact and fragmented mRNA, micro RNAs, long non‐coding RNAs, and circular RNAs (Jeppesen *et al.*, 2019; Murillo *et al.*, 2019; O'Brien *et al.*, 2020), with this ‘RNAome’ highly depending on the tissue the EVs are originating/purified from and also the isolation method (Colombo *et al.*, 2014; Simons & Raposo, 2009; Van Deun *et al*., 2014; Van Niel *et al*., 2018). Out of ∼700 genes covered by the Nanostring nCounter^®^ transcription analysis panel, we found only *INA* and *SYT1* to be associated with the col^−^ EVs and *MOG*, and *RING1* to be associated with the col^+^ EVs. Considering the mRNA is mainly found internally in the EVs, we can state that the BDEVs isolated with both approaches are mainly the same populations, and it could also be inferred that the minor proteomic changes observed are due to alterations in the EV membrane and protein corona. However, we have not examined how the enzyme‐free protocol affects the composition of EV‐associated small non‐coding RNAs, such as miRNAs, which would have completed the RNA profiling. These small nucleic acids are a prominent cargo in EVs, and their regulation and uptake can significantly influence gene expression in brain cells under both pathological and physiological conditions (Esteves *et al*., 2022; Huang, Driedonks *et al.*, 2023; Upadhya *et al.*, 2020; Wang *et al.*, 2022). They have also been described as a potential biomarker in several neurodegenerative diseases (Kumar *et al.*, 2023; Ryu *et al.*, 2023; Visconte *et al.*, 2023), likely making them crucial for a full understanding of BDEVs’ pathophysiological roles. It has been described that small ECM‐bound nanovesicles, which contain a high number of miRNAs, require enzymatic digestion to separate them from the ECM or biological scaffolds (Huleihel *et al.*, 2016). Further experiments are required to assess how a non‐enzymatic approach to isolate the BDEVs affects their miRNA content.

In conclusion, our results show that collagenase treatment leads to the degradation of several membrane proteins in BDEVs, suggesting that caution should be taken when using enzymes for EV isolation from tissues such as the brain. BDEVs isolated without collagenase show a comparable purity compared to the ones isolated with a low amount of collagenase. Moreover, isolation approaches obtained a similar EV population enrichment with just some minor proteomic differences. The relevant advantage of this enzyme‐free approach is that some key proteins at the EV membrane, among them widely accepted EV markers, are not artificially cleaved, allowing for more physiological and functional EV studies.

## AUTHOR CONTRIBUTIONS

Andreu Matamoros‐Angles: Conceptualization (equal); data curation (lead); funding acquisition (equal); investigation (equal); methodology (lead); project administration (lead); writing—original draft (lead); writing—review and editing (equal). Emina Karadjuzovic: Data curation (lead); methodology (lead); writing—original draft (equal); writing—review and editing (equal). Behnam Mohammadi: Data curation (equal); methodology (supporting); writing—original draft (supporting); writing—review and editing (equal). Feizhi Song: Data curation (supporting); methodology (supporting). Santra Brenna: Methodology (supporting); validation (supporting); writing—review and editing (supporting). Susanne C. Meister: Data curation (supporting); validation (supporting); writing—review and editing (supporting). Bente Siebels: Data curation (supporting); methodology (supporting). Hannah Voß: Data curation (supporting); formal analysis (supporting); methodology (supporting). Carolin Seuring: Data curation (supporting); methodology (supporting). Isidre Ferrer: Resources (equal). Hartmut Schlüter: Data curation (supporting); methodology (supporting). Matthias Kneussel: Writing—review and editing (supporting). Hermann C. Altmeppen: Conceptualization (supporting); methodology (supporting); writing—review and editing (equal). Michaela Schweizer: Data curation (equal); methodology (equal). Berta Puig: Investigation (supporting); methodology (supporting); writing—review and editing (equal). Mohsin Shafiq: Data curation (lead); formal analysis (lead); funding acquisition (supporting); methodology (equal); project administration (lead); writing—original draft (equal); writing—review and editing (equal). Markus Glatzel: Funding acquisition (lead); project administration (lead); resources (equal); writing—review and editing (equal).

## CONFLICT OF INTEREST STATEMENT

The authors declare no conflict of interest.

## Supporting information



Supporting information

Supporting information

Supporting information
