# Supplementary material for: Efficient enzyme‐free isolation of brain‐derived extracellular vesicles
Source: J Extracell Vesicles. 2024 Nov 7;13(11):e70011. doi: 10.1002/jev2.70011 (PMC11541858; doi:10.1002/jev2.70011)
Supplement: Supplementary file 1 — Supporting information [file JEV2-13-e70011-s003.pdf]

Supplementary Material for Matamoros-Angles A. and Karadjuzovic E. *et al.*

N°	IDs	Cohort	Sex	Age	PMI	NIA scoring	Neuropathological findings
1	**PO1**	A	M	78	2 h 15 min	A0, B0, C0	Lacunar infarcts
2	**PO2**	A	M	67	5 h	A0, B0, C0	Lacunar infarcts
3	**PO3**	A	M	71	12 h	A0, B0, C0	No neuropathological findings
4	**PO4**	A	M	61	3 h 55 min	A0, B0, C0	Metabolic encephalopathy
5	**PO5**	A	M	85	5 h 45 min	A0, B0, C0	Lacunar infarcts in striatum
6	**PO6**	A	M	64	3 h 30 min	A0, B0, C0	Mesial sclerosis
7	**PO7**	A	F	75	3 h	A0, B0, C0	Lacunar infarcts
8	**PO8**	A	F	74	2 h 45 min	A1, B1, C0	Amyloid and Tau pathology
9	**PO9**	A	F	75	4 h 55 min	A1, B1, C0	Amyloid and Tau pathology
10	**PO10**	B	F	66	3 days	A0, B0, C0	No neuropathological findings
11	**PO11**	B	F	79	4 days	A0, B0, C0	No neuropathological findings
12	**PO12**	B	F	59	>2 days	A0, B0, C0	Inflammatory profile (CD20+)
13	**PO13**	B	F	69	>2 days	A0, B0, C0	Inflammatory profile (CD8+)
14	**PO14**	B	F	68	2 days	A0, B0, C0	Encephalitis and CD8+
15	**PO15**	B	F	55	2 days	A0, B0, C0	Leukoencephalopathy

Table 1: Human frontal cortex autopsy tissues used for different BDEV preparations with collagenase-assisted and collagenase-free methods.

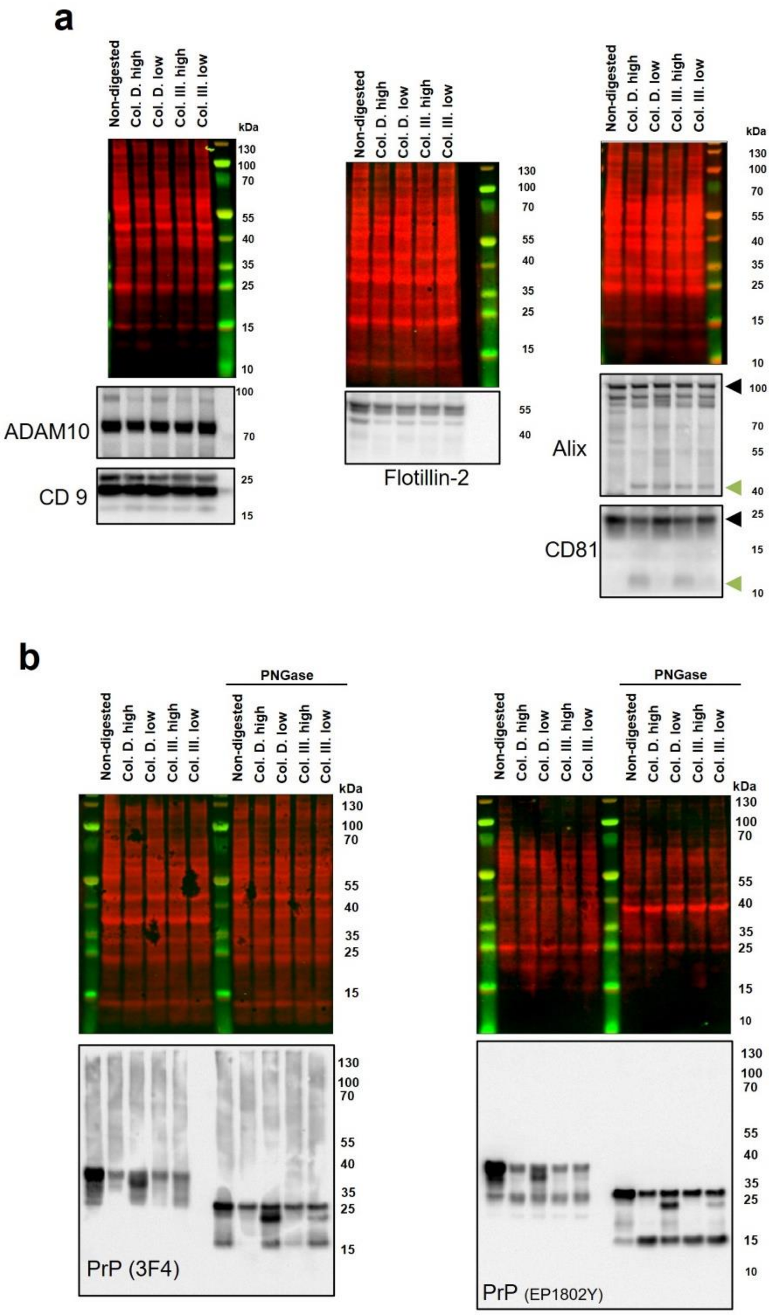

Supplementary Figure S1: Collagenase treatment effect on human brain tissue.

(a) Western blotting of human brain homogenates after the digestion with collagenase D and collagenase III. The same samples were loaded three times. CD81 and Alix presented an altered pattern but not CD9, ADAM10, or Flotillin-2. In (b), the same samples with PNGase treatment were loaded 2 times. PrP^C was detected with two antibodies, one C-terminal (EP1802Y) and the other more N-terminal (3F4). The N-glycan removal showed exacerbated PrP-C1-like, PrP-N1-like forms, and an artificial shorter full-length PrP^C, as was previously observed with mouse brain after collagenase digestion.

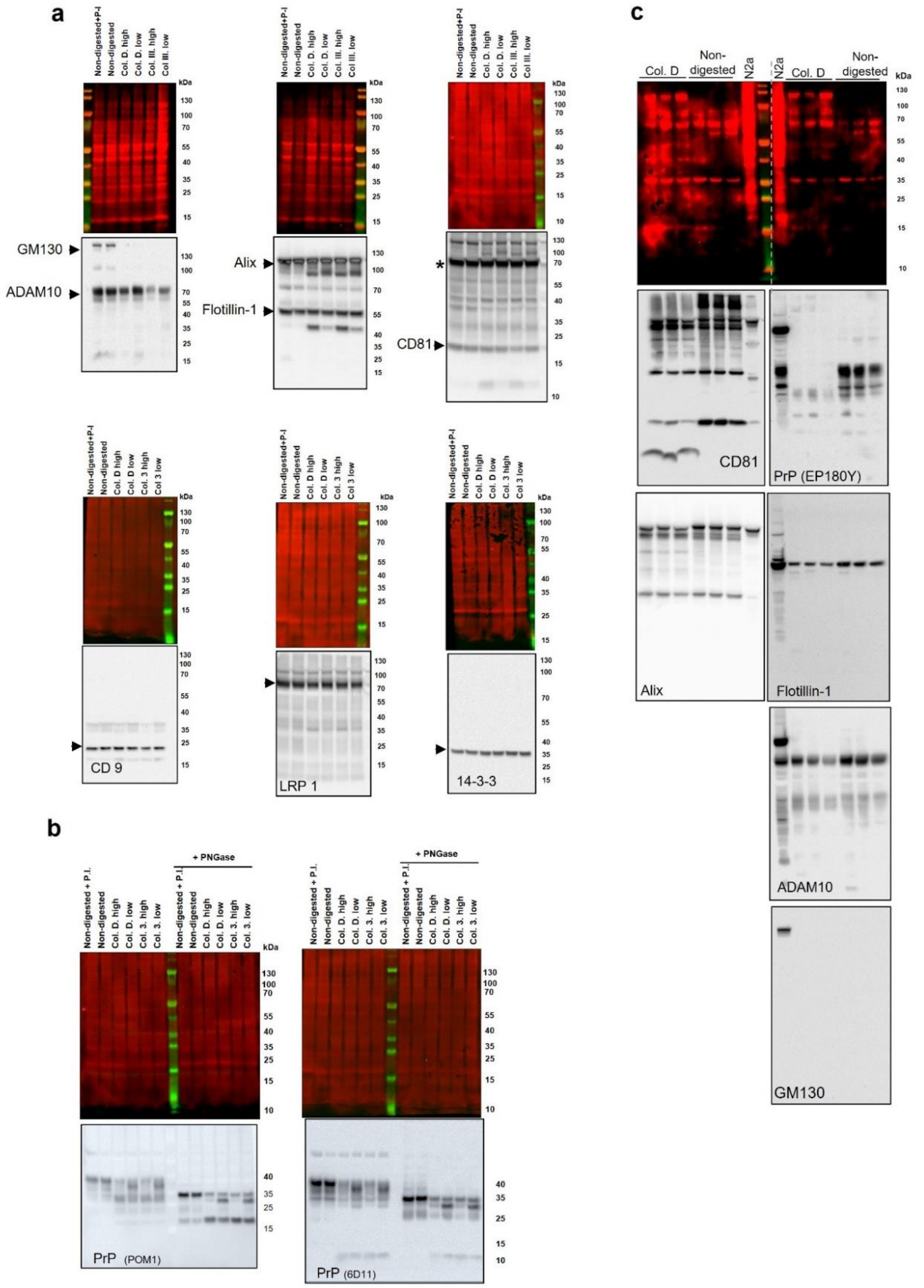

Supplementary Figure S2: Images of the uncropped blots and total protein stainings from Figure 1.

The same samples were loaded six times. Complete blots and total protein stainings of Figure 1b. GM130 was detected, and later, ADAM10 was reprobed. Alix was detected, and Flotillin-1 was reprobed. SDHA (marked as *) was detected (but not included in the final figure of the manuscript), and CD81 was reprobed. CD9, LRP1, and 14-3-3 were detected separately. (b) Complete blots and total protein staining of the western blotting of Figure 1c. Finally, in (c), the complete blots and total protein staining of Figure 1h are shown. The same samples were loaded two times. Alix was detected, and CD81 was later reprobed. On the other membrane, PrP^{C}, Flotillin-1, ADAM10 and GM130 were detected sequentially with a protein stripping in between.

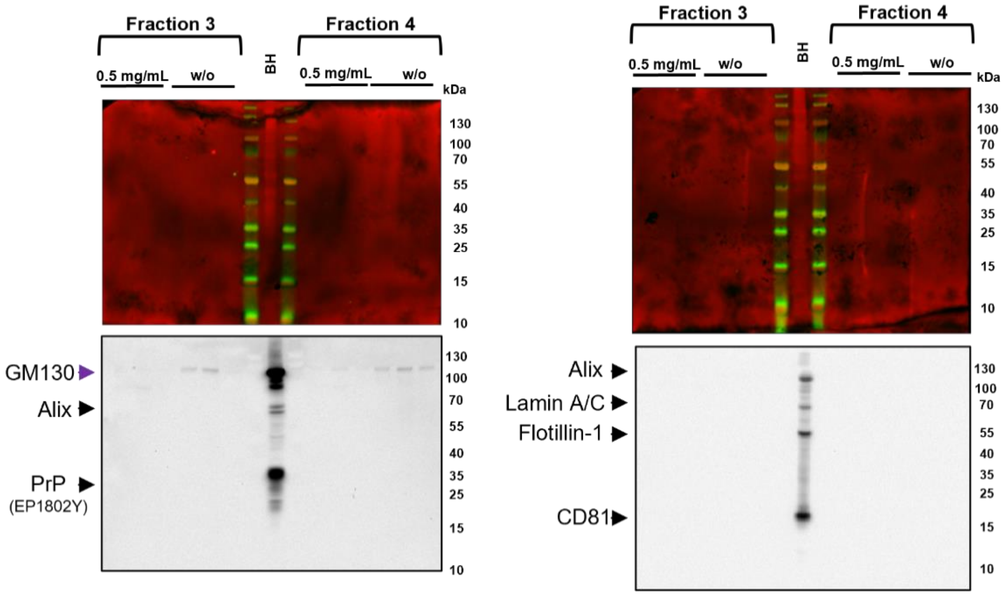

Supplementary Figure S3: EV expression markers in female mouse tissue BDEV isolation controls and fractions: 10,000*xg* pellet and pre-gradient BDEVs.

Expression of EV positive markers (Alix, CD81, and Flotillin-1), EV negative markers (Lamin A/C and GM130), and PrP^C (EP1802Y) in the BDEV isolation control samples obtained during the isolation of the BDEVs shown in Figure 3. The same samples were loaded two times. The GM130 signal was detectable in fraction 3 (F3) and fraction 4 (F4) in the samples obtained with 0 mg/mL of collagenase (w/o), but there is no expression of any other marker. The total protein staining also shows no protein expression in the samples, but it does in the total mouse brain homogenate (BH), which is used as a loading control.

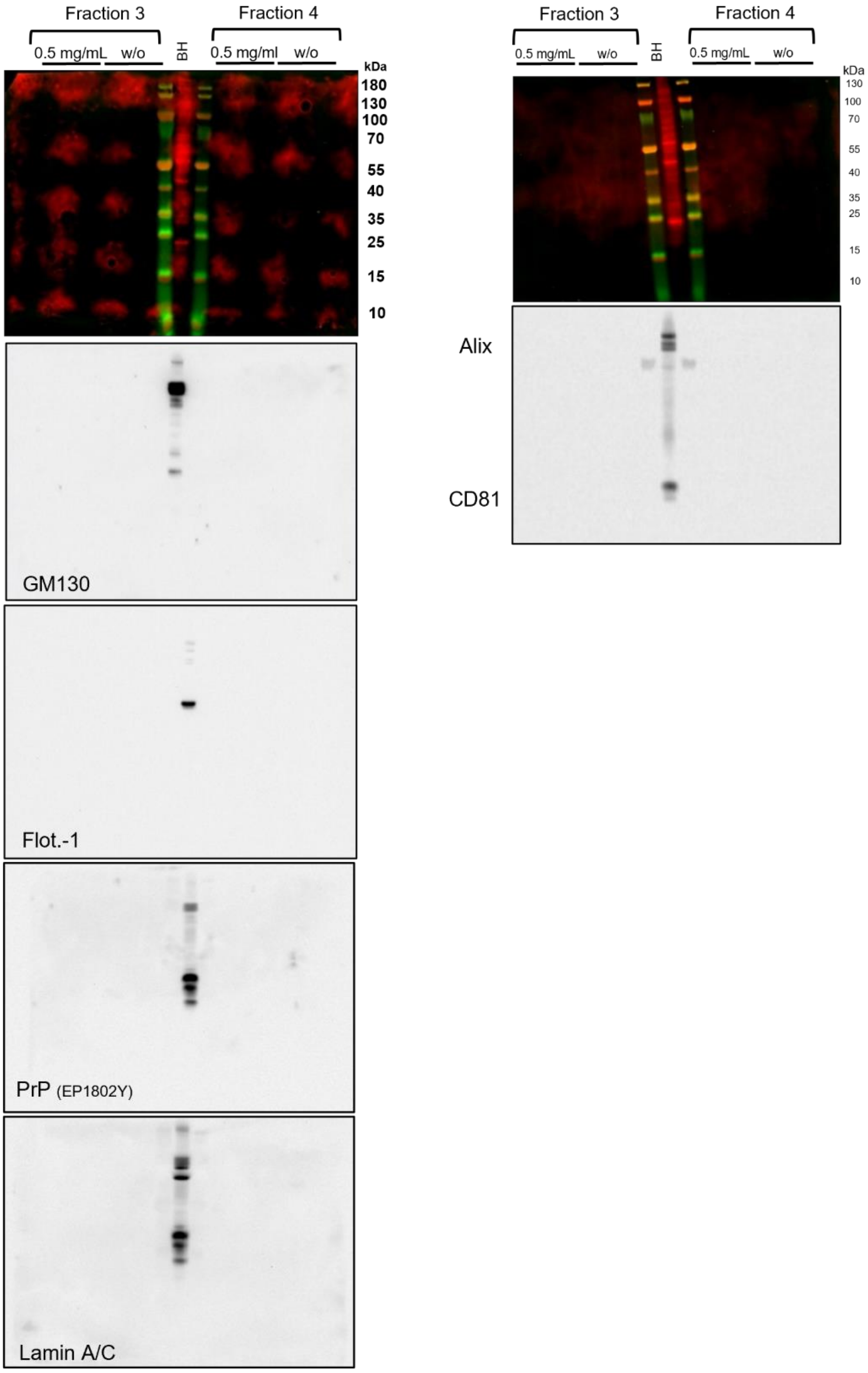

Supplementary Figure S4: EV expression markers in male human-BDEV isolation controls and fractions: 10,000*x*g pellet and pre-gradient BDEVs.

Expression of EV positive markers (Alix, CD81, and Flotillin-1), EV negative markers (Lamin A/C and GM130), and PrP^C (EP1802Y) in the BDEV isolation control samples obtained during the isolation of the BDEVs shown in Figure 4. The same samples were loaded two times. The fraction 3 (F3) and fraction 4 (F4) showed no expression of any marker. The total protein staining also shows no protein expression in the samples, but it does in the total human brain homogenate (BH) used as a loading control.

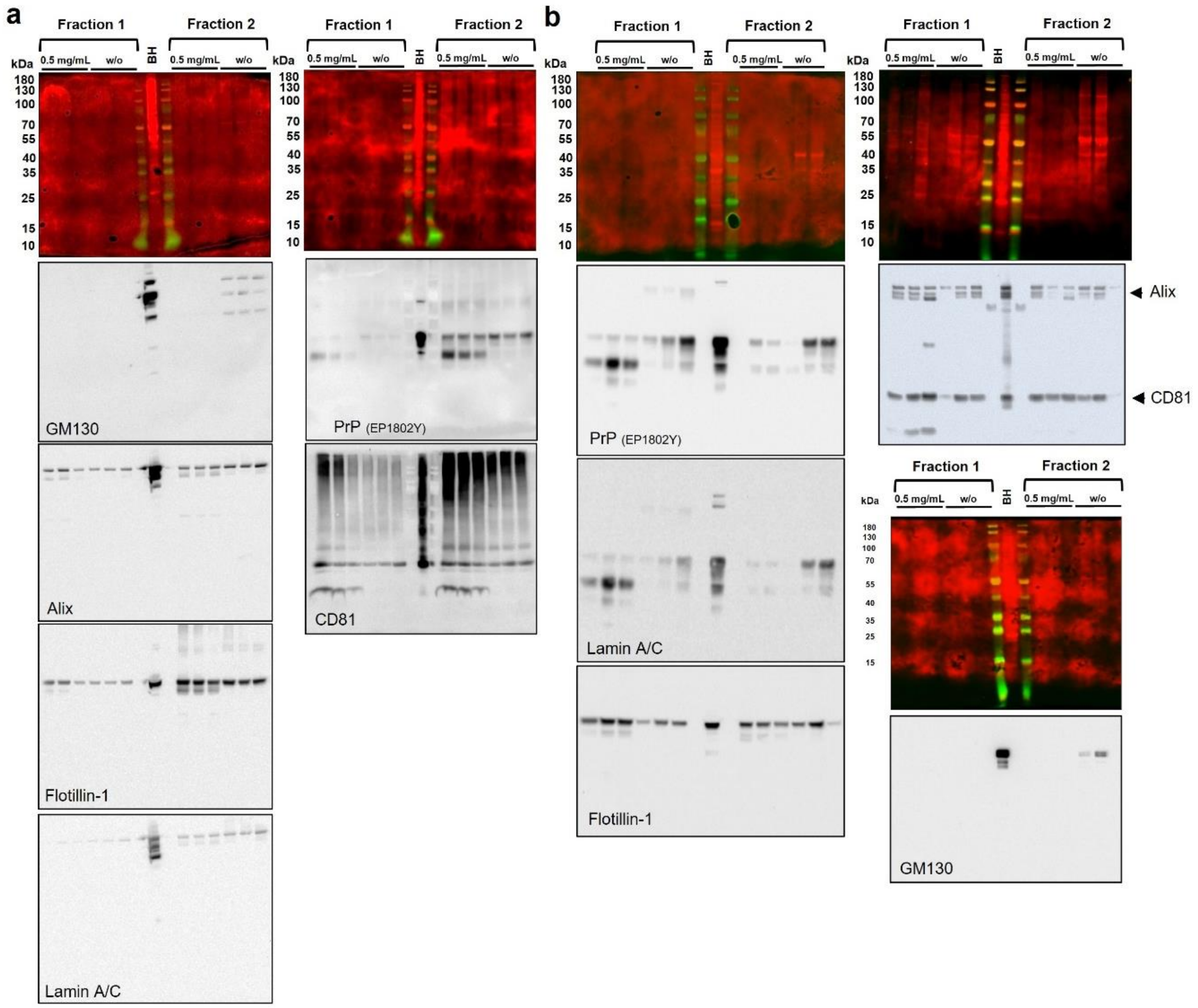

Supplementary Figure S5: Images of the uncropped blots and total protein stainings from Figure 3 and Figure 4. (a) The corresponding uncropped films from Figure 3 were the same samples that were loaded twice. GM130 was initially detected, followed by an intercalated stripping, Alix, Flotillin-1, and Lamin A/C were detected. On the other membrane, PrP^{C} (using EP1802Y antibody) was detected, and then CD81 was reprobed. (b) The same samples were loaded three times with their corresponding uncropped films in Figure 4a. PrP^{C} was initially detected in the first membrane, and later, after an intercalated stripping, Flotillin-1 and Lamin A/C were detected. On the other membrane, first, Alix was detected, and then CD81 was reprobed. Finally, GM130 was detected alone on the last membrane.

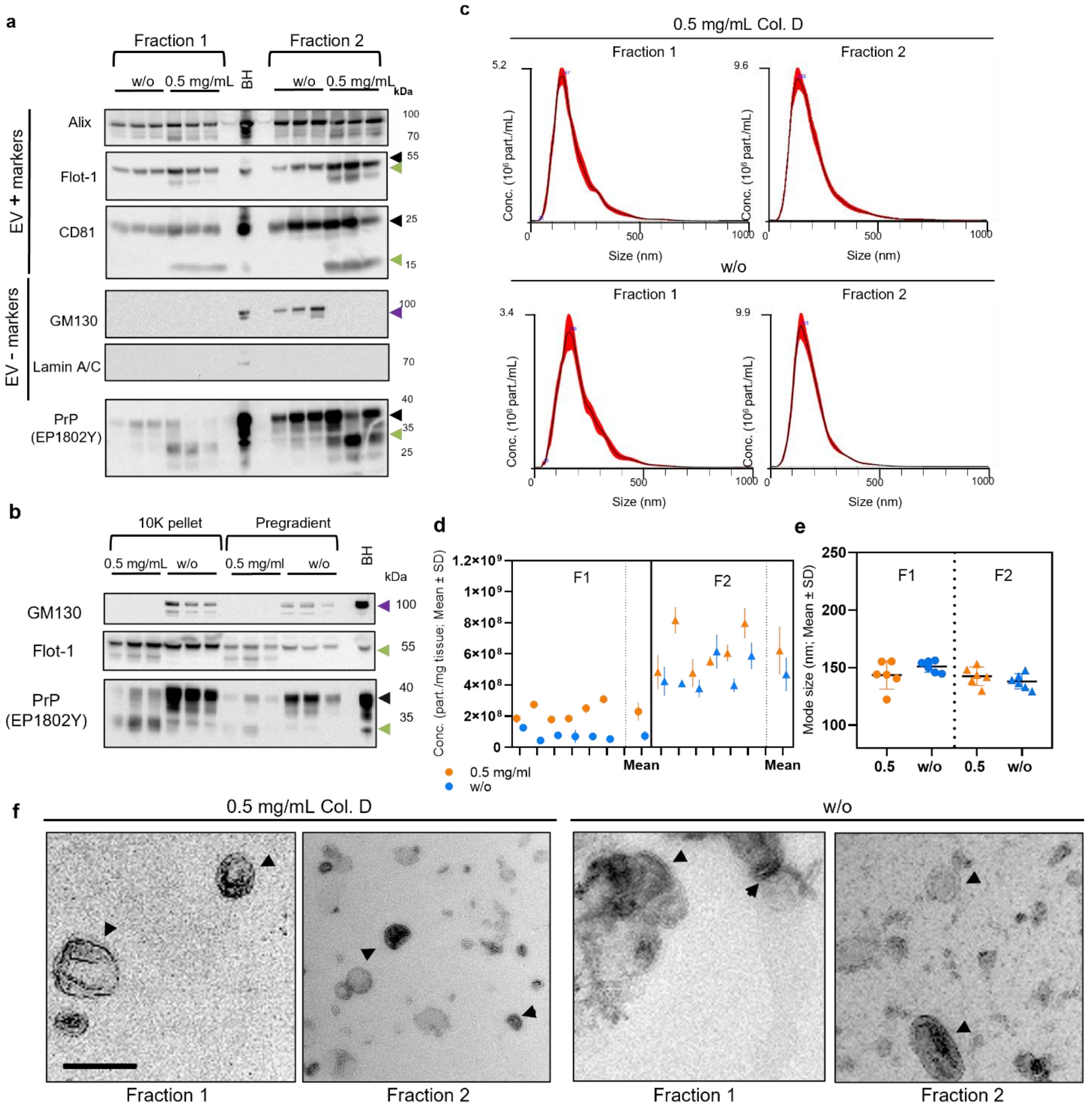

Supplementary Figure S6: BDEV isolation from male mouse brain tissue without enzymatic digestion maintains BDEV purity and prevents artificial proteolytic processes as in female samples.

(a) Western blots of F1 and F2 BDEVs samples isolated from male mouse brain tissue, with or without the addition of 0.5 mg/mL of collagenase D (n = 3 for each condition), were labeled for PrP^{C}, the EV positive markers Alix, Flotillin-1, and CD81 as well as for the Golgi and nucleus markers, GM130 and Lamin A/C as EV negative markers. The green arrowheads indicate the artificial cleavage observed in the proteins PrP^{C}, CD81, and Flotillin-1, while the purple arrowhead denotes the unexpected presence of GM130. A total mouse brain homogenate (BH) served as a control. In (b), the 10,000xg pellet (10K) and the pregradient BDEVs showed a GM130 total disappearance when collagenase was used, and PrP^{C} and Flotillin-1 displayed the cleavage pattern observed in the BDEVs. (c) Representative size distribution graphs from NTA analysis of F1 and F2 BDEVs show the expected normal-like distribution. The concentration of particles per mg of initial tissue (d) and mode size analysis in nm (e) of BDEVs obtained with both protocols were measured with NTA (n = 6 for each condition). The particle concentration values are shown as the mean value of each sample measurement with its SD derived from its technical replicates. No differences in the particle mode size were observed between the same fraction in both protocols, but the $F1^{-}$ displayed significantly fewer particles/mg of tissue than the $F2^{+}$. However, in both protocols, the F2 had significantly more particles than the F1. (f) TEM images of negative stained BDEV showing the typical double membrane and cup shape. BDEVs are indicated with arrowheads. Scale bar = 200 nm. Data are presented as mean ± S.D in (d) and (e).

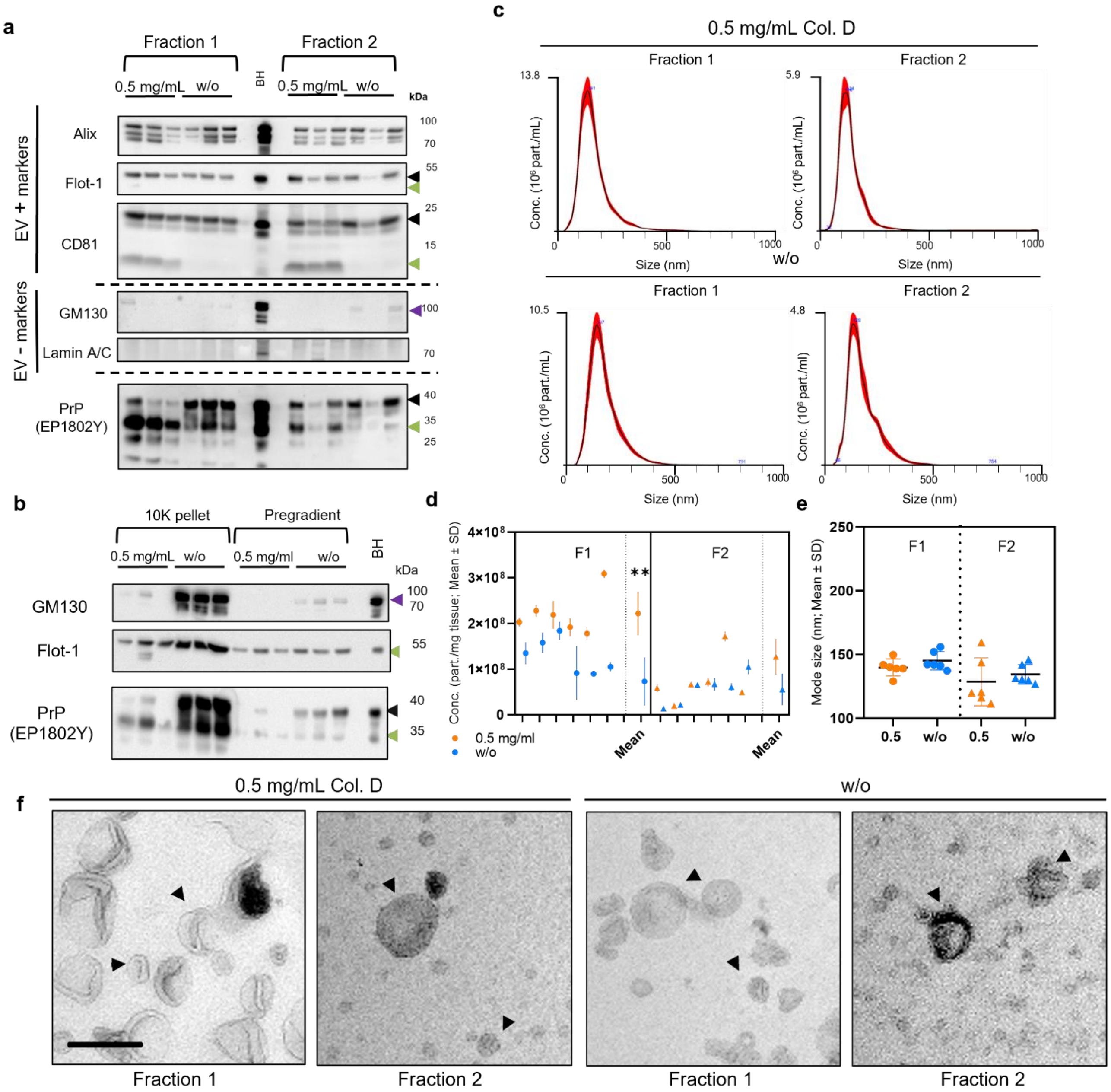

Supplementary Figure S7: BDEV isolation from female human brain tissue without enzymatic digestion maintains BDEV purity and prevents artificial proteolytic processing.

Representative western blots of F1 and F2 BDEVs samples isolated from female human brain tissue, with or without the addition of 0.5 mg/mL of collagenase D (*n* = 3 for each condition), for PrP^C, the EV positive markers Alix, Flotillin-1, and CD81 and the Golgi and nucleus markers, GM130 and Lamin A/C, as EV negative markers. The green arrowheads indicate the artificial cleavage observed in the proteins PrP^C, CD81, and Flotillin-1. A total human brain homogenate (BH) was used as a loading control. In (b), the 10,000*xg* pellet (10K) and the pregradient BDEVs showed a GM130 total disappearance when collagenase was used, and PrP^C and Flotillin-1 displayed the cleavage pattern observed in the BDEVs. (c) Representative size distribution graphs from NTA analysis of F1 and F2 BDEVs show the expected normal-like distribution. The concentration of particles per mg of initial tissue (d) and mode size analysis in nm (e) of BDEVs obtained with both protocols were measured with NTA (*n* = 6 for each condition). The particle concentration values are shown as the mean value of each sample measurement with its SD derived from its technical replicates. No differences were observed in the particle concentration between both protocols in F2, however, the $F1^+$ had significantly more particles than the other groups F1-. No differences are observed regarding the particle size. (f) TEM images of negative stained BDEV illustrating the typical double membrane and cup shape. BDEVs are indicated with arrowheads. Scale bar = 200 nm. Data are presented as mean ± S.D. in (d) and (e).

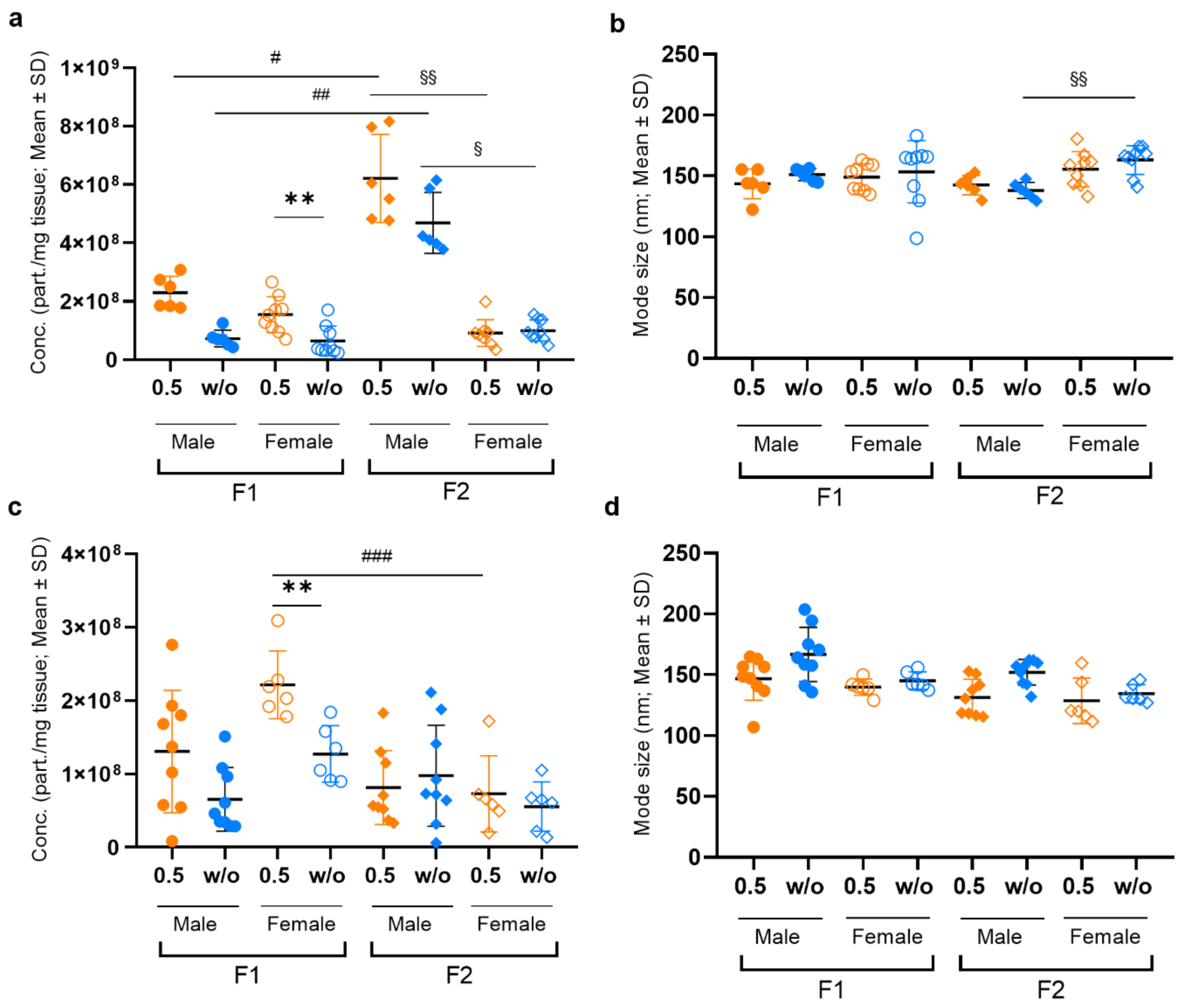

Supplementary Figure S8: The NTA analysis revealed comparable BDEV concentration and size between samples from males and females, both in mouse and human-derived samples.

Concentration and size of particles derived from mouse (male and female) (a and b) and human (male and female) (c and d) tissues obtained with 0.5 mg/ml collagenase or without (w/o). F1^{+} has significantly more particles than F1- just in the female-derived samples (**p < 0.01). In male mouse-derived samples, there are more particles in F2 than in F1 in both protocols; opposite phenomenon is observed in the female F2+, which had less particles than the F1 ($^{\#}p < 0.05$ and $^{\#\#}p <$ 0.01). Comparing the same fraction isolated with the same approach between sexes (in the mouse case), more particles are observed in the male F2 compared to the female F2, and the F2- female samples are more significant than the F2- male samples ($^{\S}p < 0.05$ and $^{\S\S}p < 0.01$). Data rearranged from Figures 3, 4, S6 and S7. Data is presented as mean ± S.D.

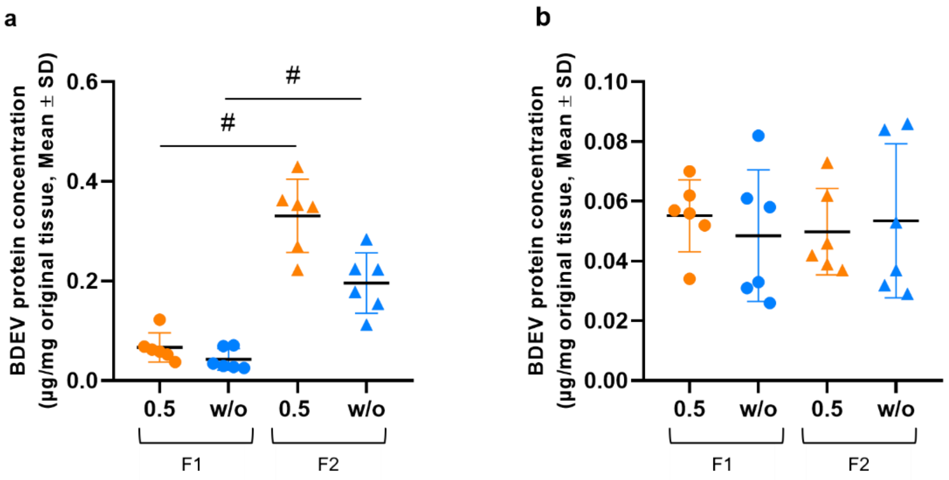

Supplementary Figure S9: The protein concentration of the BDEV samples isolated with and without collagenase is not different in human and mouse samples.

Protein concentrations of F1 (circle) and F2 (triangle) BDEV samples from male mouse (a) and female human (b) tissues isolated with 0,5 mg/ml of collagenase (orange) or without (blue). In both cases, no differences in the protein expression were observed between both approaches in the same fraction. As seen on the NTA, the F2 in male mouse samples presented significantly more protein than the respective F1 ($^{\#}p < 0.05$). Data are presented as mean ± S.D.

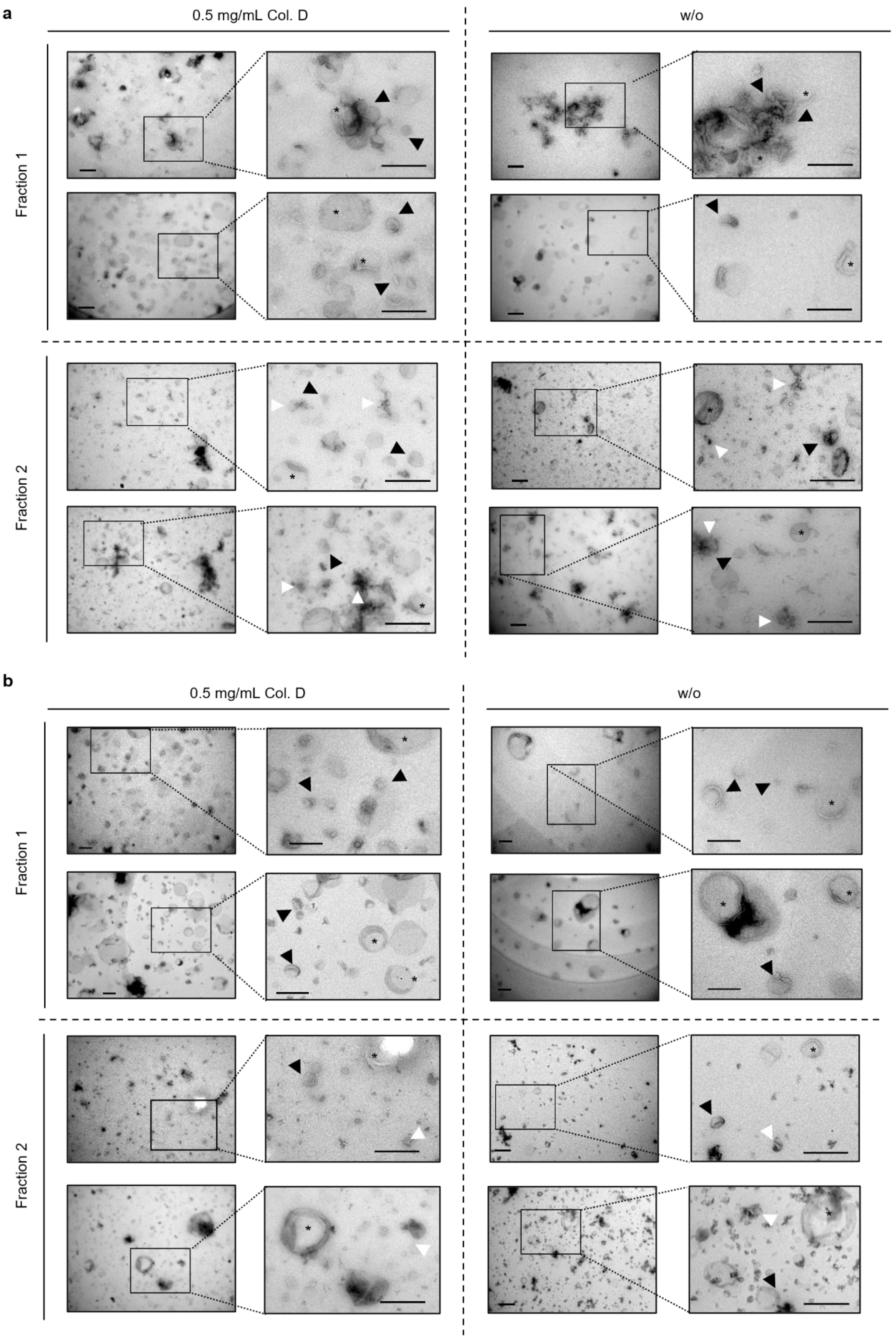

Supplementary Figure S10: Extended BDEVs morphology description by TEM.
TEM images of female mouse (a) and male human (b) BDEVs obtained with (0.5 mg/mL) or without (w/o) collagenase. Detailed membranous BDEV structures are observed in all the samples (arrowheads), and also large vesicles folded (asterisk). In both cases, as expected, the F2 shows more debris and aggregated structures. Scale bars = 500 nm.

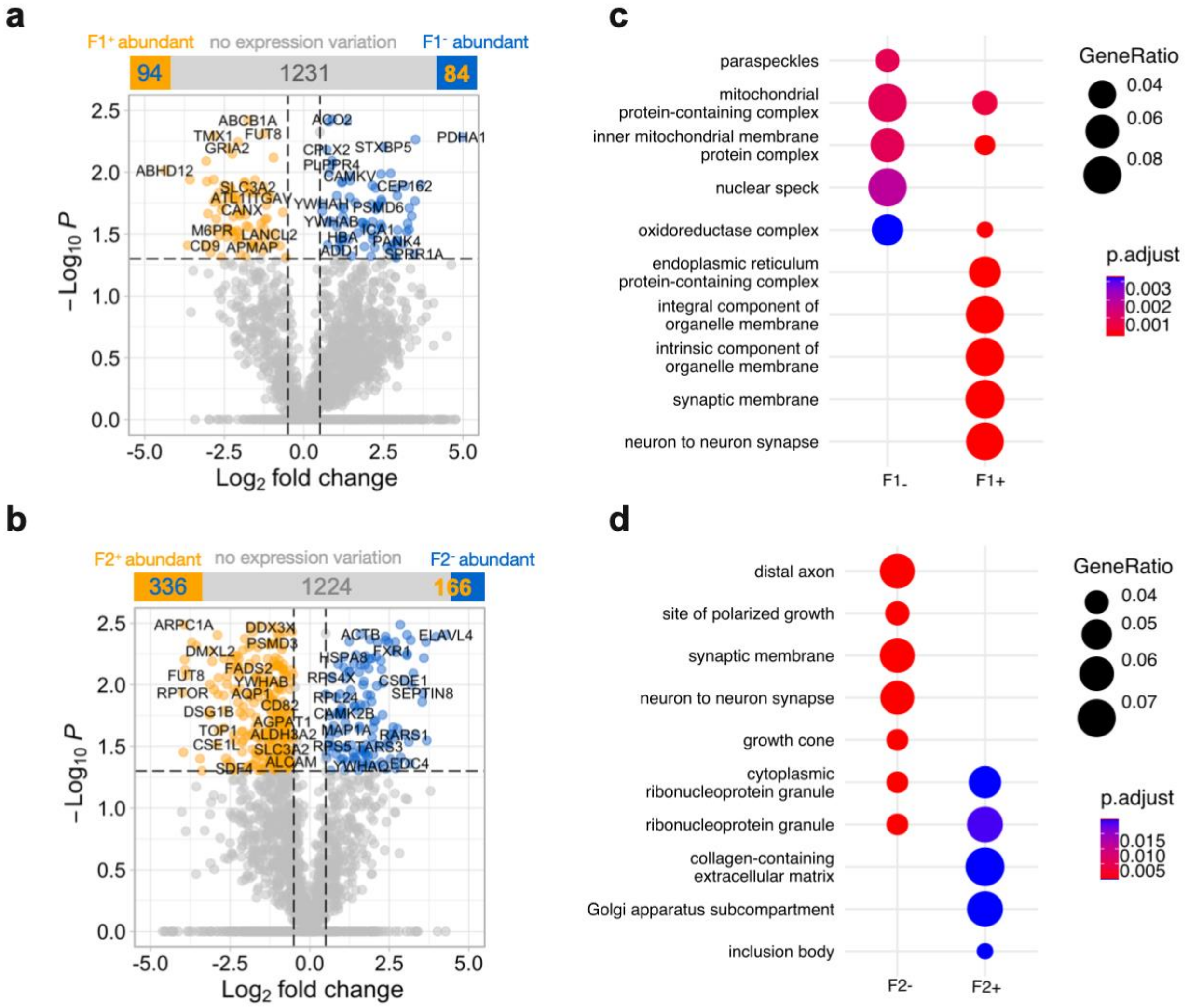

Supplementary Figure S11: Comparative enrichment analysis highlights minor proteomic differences in the col+ and col- EVs isolated from mice brain.

(a) Volcano plot showing proteins relatively down (shown in orange) and up-regulated (shown in blue) in F1$^-$ compared to F1$^+$. 84 proteins were found to be overexpressed, and 94 proteins were found to be downregulated in F1$^-$ compared to F1$^+$ (b) Volcano plot showing proteins relatively under-represented (shown in orange) and up-regulated protein (shown in blue) in F2$^-$. 166 proteins were found to be more abundant, and 336 proteins were found to be less expressed in F2$^-$ when compared to F2$^+$. The intercepts along the x-axis represent the thresholds for expression changes: -1.5-fold for down-regulation and +1.5-fold for up-regulation. Meanwhile, the intercept on the y-axis indicates the threshold set for a p-value of 0.05. (c) Dot plot showing the GO terms exclusively associated to the proteomes of F1$^-$ and F1$^+$ BDEVs. (d) Dot plot showing the GO terms exclusively associated to the proteomes of F2$^-$ and F2$^+$ BDEVs. Size of the dots is a measure of the Gene ratio for each displayed enrichment term (ratio of number of proteins associated to the certain GO term, total number of the differentially regulated proteins of that group). GO terms adjusted p-value is represented by the color gradient; color key included in the figure.

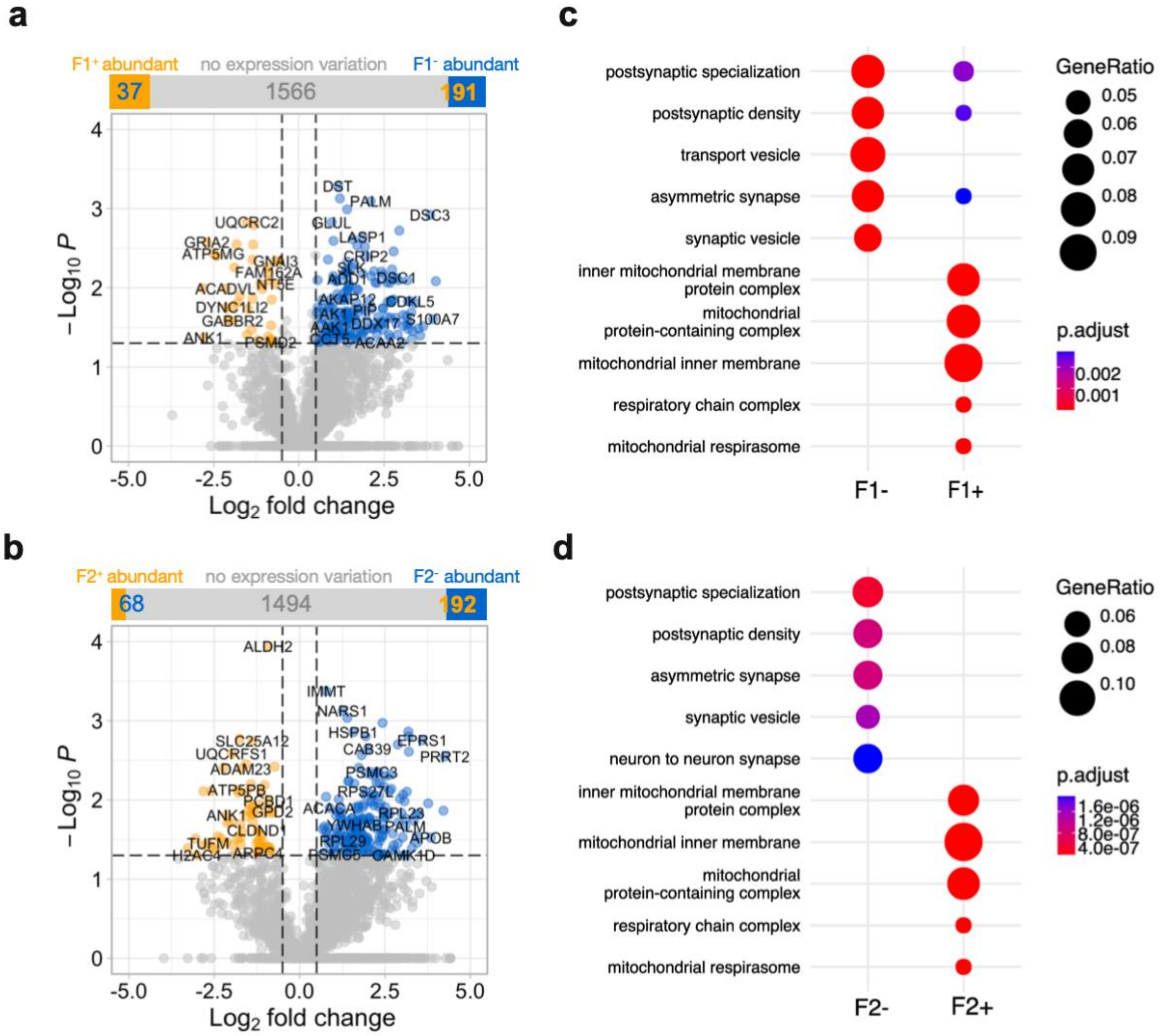

Supplementary Figure S12: Comparative enrichment analysis highlights minor proteomic differences in the col+ and col- EVs isolated from human brain.

(a) Volcano plot showing proteins relatively under-represented (displayed in orange) and up-regulated in F1$^-$ (displayed in blue). 191 proteins were found to be more abundant, and 37 proteins were found to be less expressed in F1$^-$, as shown in the column graph associated with the volcano plot. (b) Volcano plot showing proteins relatively down (displayed in orange) and up-regulated in F2$^-$ EVs (displayed in blue). 192 proteins were found to be increased, and 68 proteins were found to be decreased in F2$^-$, shown in the column graph associated with the volcano plot. The intercepts along the x-axis represent the thresholds for expression changes: -1.5-fold for down-regulation and +1.5-fold for up-regulation. Meanwhile, the intercept on the y-axis indicates the threshold set for a *p*-value of 0.05. (c) Dot plot showing the GO terms exclusively associated to the proteomes of F1$^-$ and F1$^+$ BDEVs. (d) Dot plot showing the GO terms exclusively associated to the proteomes of F2$^-$ and F2$^+$ BDEVs. Size of the dots is a measure of the Gene ratio for each displayed enrichment term (ratio of number of proteins associated to the certain GO term, total number of the differentially regulated proteins of that group). GO terms adjusted p-value is represented by the color gradient; color key included in the figure.
